# Drp1 is widely, yet heterogeneously, distributed in the mouse central nervous system

**DOI:** 10.1186/s13041-020-00628-y

**Published:** 2020-06-10

**Authors:** Ting-Ting Luo, Chun-Qiu Dai, Jia-Qi Wang, Zheng-Mei Wang, Yi Yang, Kun-Long Zhang, Fei-Fei Wu, Yan-Ling Yang, Ya-Yun Wang

**Affiliations:** 1 National Demonstration Center for Experimental Preclinical Medicine Education, Air Force Medical University (The Fourth Military Medical University), Xi’an, 710032 China; 2 Mental Health Center, West China Hospital of Sichuan University, Chengdu, 610041 China; 3 Third Medical District, Lintong Rehabilitation and Convalescent Centre, Xi’an, 710600 China; 4 Medical College of Yan’an University, Yan’an, 716000 China; 5 Department of Rehabilitation Physiotherapy, Xi-Jing Hospital, Air Force Medical University (The Fourth Military Medical University), Xi’an, 710032 China; 6 Department of Hepatobiliary Surgery, Xi-Jing Hospital, Air Force Medical University (The Fourth Military Medical University), Xi’an, 710032 China

## Abstract

**Objectives:**

Drp1 is widely expressed in the mouse central nervous system and plays a role in inducing the mitochondrial fission process. Many diseases are associated with Drp1 and mitochondria. However, since the exact distribution of Drp1 has not been specifically observed, it is difficult to determine the impact of anti-Drp1 molecules on the human body. Clarifying the specific Drp1 distribution could be a good approach to targeted treatment or prognosis.

**Methods:**

We visualized the distribution of Drp1 in different brain regions and explicated the relationship between Drp1 and mitochondria. GAD67-GFP knock-in mice were utilized to detect the expression patterns of Drp1 in GABAergic neurons. We also further analyzed Drp1 expression in human malignant glioma tissue.

**Results:**

Drp1 was widely but heterogeneously distributed in the central nervous system. Further observation indicated that Drp1 was highly and heterogeneously expressed in inhibitory neurons. Under transmission electron microscopy, the distribution of Drp1 was higher in dendrites than other areas in neurons, and only a small amount of Drp1 was localized in mitochondria. In human malignant glioma, the fluorescence intensity of Drp1 increased from grade I-III, while grade IV showed a declining trend.

**Conclusion:**

In this study, we observed a wide heterogeneous distribution of Drp1 in the central nervous system, which might be related to the occurrence and development of neurologic disease. We hope that the relationship between Drp1 and mitochondria may will to therapeutic guidance in the clinic.

## Introduction

Drp1 (Dynamin-related protein) is an ~ 80-kDa protein (monomer) that is widely expressed in the brain, lung, heart, kidney, spleen, liver, hepatocytes, testis and fibroblasts in humans [1, 2]. Drp1 contains an N-terminal GTPase domain, a helical domain at the center and a GED (GTPase effector domain) at the C-terminus [3]. In the cytoplasm, Drp1 exists as a dimer or tetramer and functions to induce the mitochondrial fission process [4, 5]. Mitochondria are organelles that are responsible for several vital cell functions, including respiration, oxidative phosphorylation, and regulation of apoptosis [6]. The brain is an organ that requires a high energy level. In the brain, mitochondria move along cytoskeletal tracks to sites of high energy demand, such as synapses, and change their morphology by fusion and fission in response to cellular metabolic activity [7]. Therefore, the balance of mitochondrial fission and fusion under the control of Drp1 is significant in maintaining brain function and energy supply [8]. Drp1 overexpression or mutation can alter this balance. Mutant Drp1 causes mitochondria to collapse into perinuclear clusters that contain a highly interconnected network [4, 9]. Additionally, lack of Drp1 results in mitochondrial elongation and connection of mitochondrial tubules [10]. These elongated mitochondria gradually accumulate oxidative damage and transform from elongated tubules into large spheres [11]. Such changes will finally lead to nervous system diseases.

It has been confirmed that many diseases are related to Drp1 and mitochondria, including neurodegenerative diseases and neuropathic pain [12]. Gao et al. have demonstrated that mitochondrial dysfunction is a common prominent early pathological feature in neurodegenerative diseases [13]. A large number of studies have demonstrated that mitochondrial dysfunction is one of the best documented abnormalities and prominent early features in brain neurodegenerative diseases. Conversely, Guo et al. demonstrated that mitochondrial fission leads to an increase in ROS [14], and the increase in ROS will further induce neuropathic and inflammatory pain [15]. Ferrari et al. found that in models of chemotherapy-induced neuropathic pain, ROS greatly induces Drp1-dependent mitochondrial fission [16]. To identify the target treatment strategy, some researchers have identified certain molecules as Drp1 inhibitors, including P110 and mdivi-1 [16, 17]. However, the impact of these molecules on the human body and their range of functions are still unclear.

In addition to neurodegenerative diseases and neuropathic pain, glioma is also correlated with Drp1-mediated changes in mitochondrial dynamics. Eugenio-Pérez et al. showed that Drp1 and mitochondrial dynamics are involved in the pluripotency maintenance of glioma stem cells. Additionally, Drp1 upregulation can support glioma cells to survive in circumstances far from the vasculature and lacking nutrients. Therefore, Eugenio-Pérez et al. raised the point that Drp1 and mitochondria contribute to gliomagenesis under cell homeostasis disorder [18]. Nevertheless, from the aspect of glioma treatment and prognosis, it remains to be determined whether there is a correlation between the glioma grade and Drp1 expression changes. Moreover, antineoplastic drug development of Drp1 also requires information on the Drp1 distribution and level changes.

Currently, the Drp1 subcellular distribution between cytoplasm and mitochondria has been observed in HeLa cell lines in several investigations [19]. Despite prior knowledge that Drp1 is widely expressed in organs such as the brain from the macro perspective, the exact distribution of Drp1 in different neuronal compartments or even in the central nervous system is still lacking experimental evidence [1, 19]. Thus, an understanding of the exact distribution of Drp1 in the central nervous system is urgently required. Clarifying the specific Drp1 distribution and Drp1 expression changes in glioma could be a good approach to the targeted treatment or prognosis for diseases.

In this study, we investigated the specific distribution of Drp1 in neurons, GABAergic (γ-aminobutyric acid) neurons and mitochondria under an optical microscope and TEM (transmission electron microscope). We also explored the changes in expression of Drp1 in grade I-IV human malignant glioma. Taking together the above results, we conclude that Drp1 is widely but heterogeneously distributed in the central nervous system, and this heterogeneous distribution may contribute to the occurrence and development of neurologic diseases. We hope that this research may provide novel insights into targeted treatment of disease in the clinic.

## Results

### Drp1 is widely distributed in the central nervous system

To explore the specific distribution of Drp1 in the mouse CNS (central nervous system), we observed its expression in different mouse brain regions and the spinal cord.

### At the protein level

**Fig. 1:**
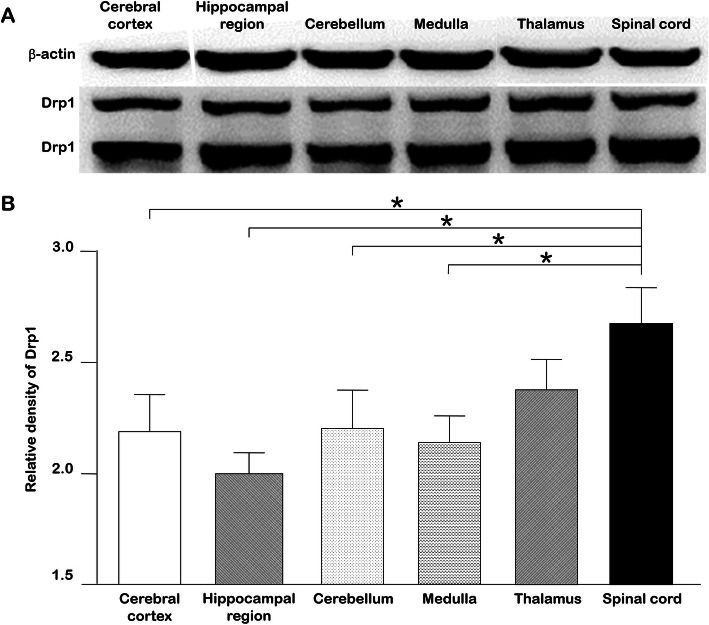
Drp1 protein content in the brain and spinal cord. **a**. Western blot results for the cortex, hippocampal region, cerebellum, medulla, thalamus and spinal cord. **b**. Relative density of Drp1. ImageJ software was used to calculate the gray value of each band, the ratio of the gray value of Drp1/β-actin was calculated, and one-way ANOVA was used for statistical analysis. The results showed no significant difference in the expression of Drp1 in the six regions (*p* > 0.05). Thus, we further utilized LSD (least significant difference) to compare each group. The expression of Drp1 was higher in the spinal cord than in the cerebral cortex, hippocampal region, cerebellum and medulla (*p* = 0.043, 0.046, 0.009, 0.028, respectively); however, there was no difference in the expression of Drp1 among different brain nuclei (*p* > 0.05). * *p* < 0.05

To preliminarily explore a potential regional distribution of Drp1 protein, western blot was used to detect the protein in the cortex, hippocampal region, cerebellum, medulla, thalamus and spinal cord. The results showed differences in Drp1 at the protein level in six regions, with the highest expression in the spinal cord and lowest expression in the cortex, hippocampal region and cerebellum (*p* < 0.05) (Fig. 1).

We next explored the exact expression of Drp1 utilizing immunofluorescence staining. Drp1 expression was determined based on the labeling intensity, which was classified as no signal (−), low signal (+), moderate signal (++), high signal (+++), and strong signal (++++) [20].

**Fig. 2:**
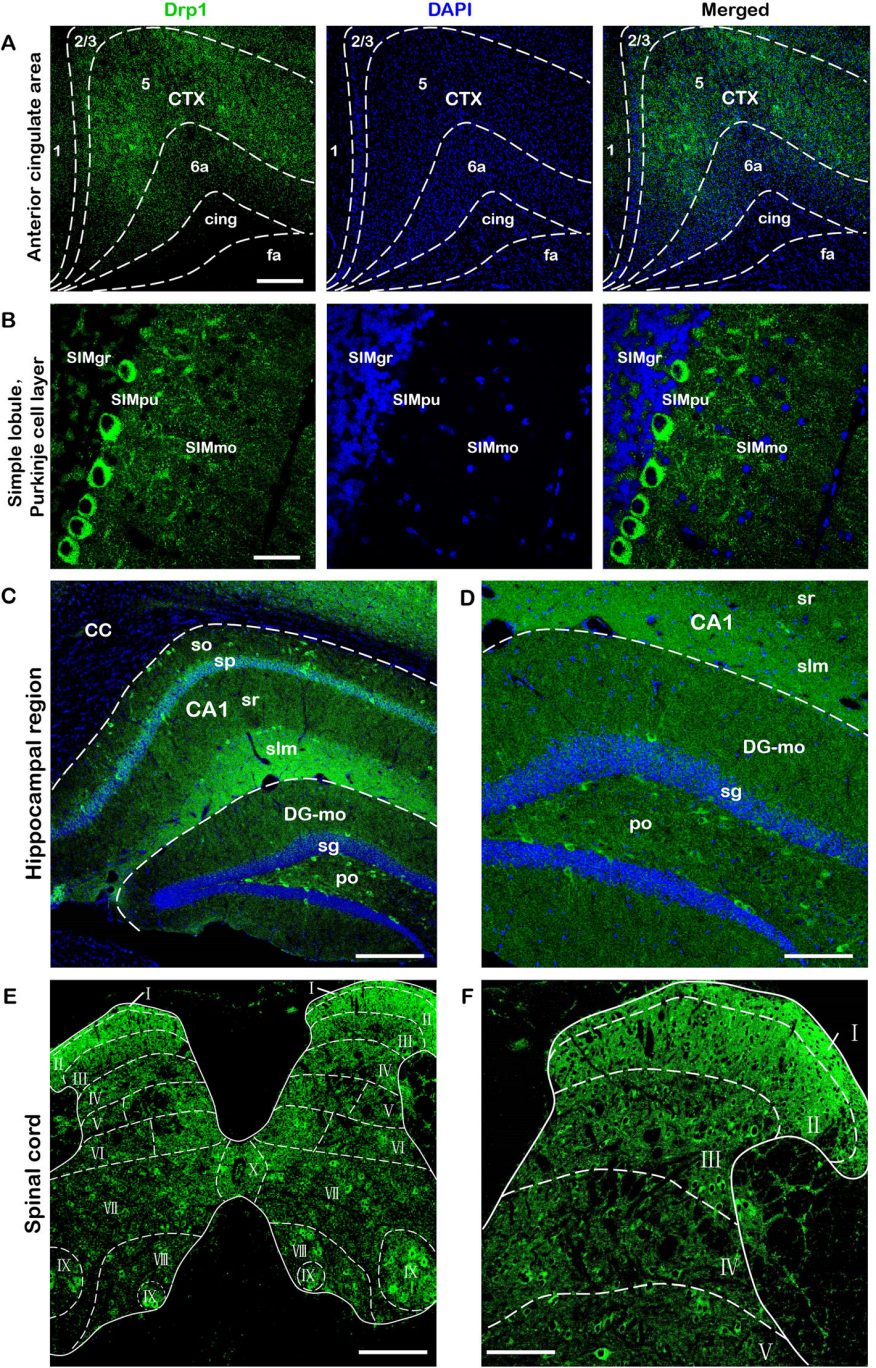
Drp1 protein distribution in the cerebral cortex, cerebellum, hippocampal region and spinal cord. **a**. The distribution of Drp1 in the cerebral cortex. Drp1 was barely expressed in the first layer of the CTX, and specific staining results were obtained in the second/third layer. Compared with the other layers, Drp1 expression was higher in the fifth layer than the others. **b**. The distribution of Drp1 in the cerebellum. Drp1 was expressed in the cytoplasm around the nucleus (DAPI). In addition, Drp1-positive protuberant structures were also found in the mo region, which were long strips and, possibly, axons. **c**. The distribution of Drp1 in the hippocampal region. The distribution of Drp1 (green) and DAPI (blue) in the CA1 and DG regions. Drp1 was expressed at low levels in CA1, DG-mo and DG-sg. Drp1 was highly expressed in DG-po. **d**. The magnified image of C. From D, we can see that some Drp1-positive cells in DG-mo and DG-po regions had a protuberance around them, which might represent axons or dendrites. **e**. Drp1 distribution in the spinal cord. **e** provides a complete view of the spinal cord. In the deep layers (VII, VIII, IX, X), Drp1 expression was slightly higher than in the superficial layers (I, II, III). **f**. In the spinal dorsal horn, Drp1 was weakly expressed. Bar = 200 μm **a, c, e**; 100 μm **d, f**; 30 μm **b**. Abbreviations: CTX: cerebral cortex; cing: cingulum bundle; SIMpu: simple lobule, Purkinje cell; SIMmo: simple lobule, molecular layer; SIMgr: simple lobule, granular layer; cc: corpus callosum; CA: Ammon’s horn; CA1-so: field CA1, stratum oriens; CA1-sp: field CA1, pyramidal layer; CA1-sr: field CA1, stratum radiatum; CA1-slm: field CA1, stratum lacunosum-moleculare; DG-mo: molecule layer of dentate gyrus; DG-sg: granular cell layer of dentate gyrus; DG-po: polymorphic layer of dentate gyrus

The results showed that Drp1 was expressed in layer 5 and 6a of the cerebral cortex, and some expression was also observed in layer 2, although we did not detect obvious Drp1 expression in layer 1 (Fig. 2 a). Drp1 was poorly expressed in the hippocampal region. However, some neurons in the DG-po (polymorphic layer of dentate gyrus) and CA1so (field CA1, stratum oriens) displayed Drp1 expression in the soma and neurite (Fig. 2 c, d). In the cerebellum, Drp1 protein expression was mainly concentrated in the SIMpu (simple lobule, Purkinje layer). Drp1-positive cells in pu were arranged in a single layer, separating the mo (molecular layer) and the gr (granular layer) (Fig. 2 b). In the spinal cord, high Drp1 expression was observed in the deep layers (VII, VIII, IX, X), which was slightly higher than that in the superficial layers (I, II, III) (Fig. 2 e, f).

**Fig. 3:**
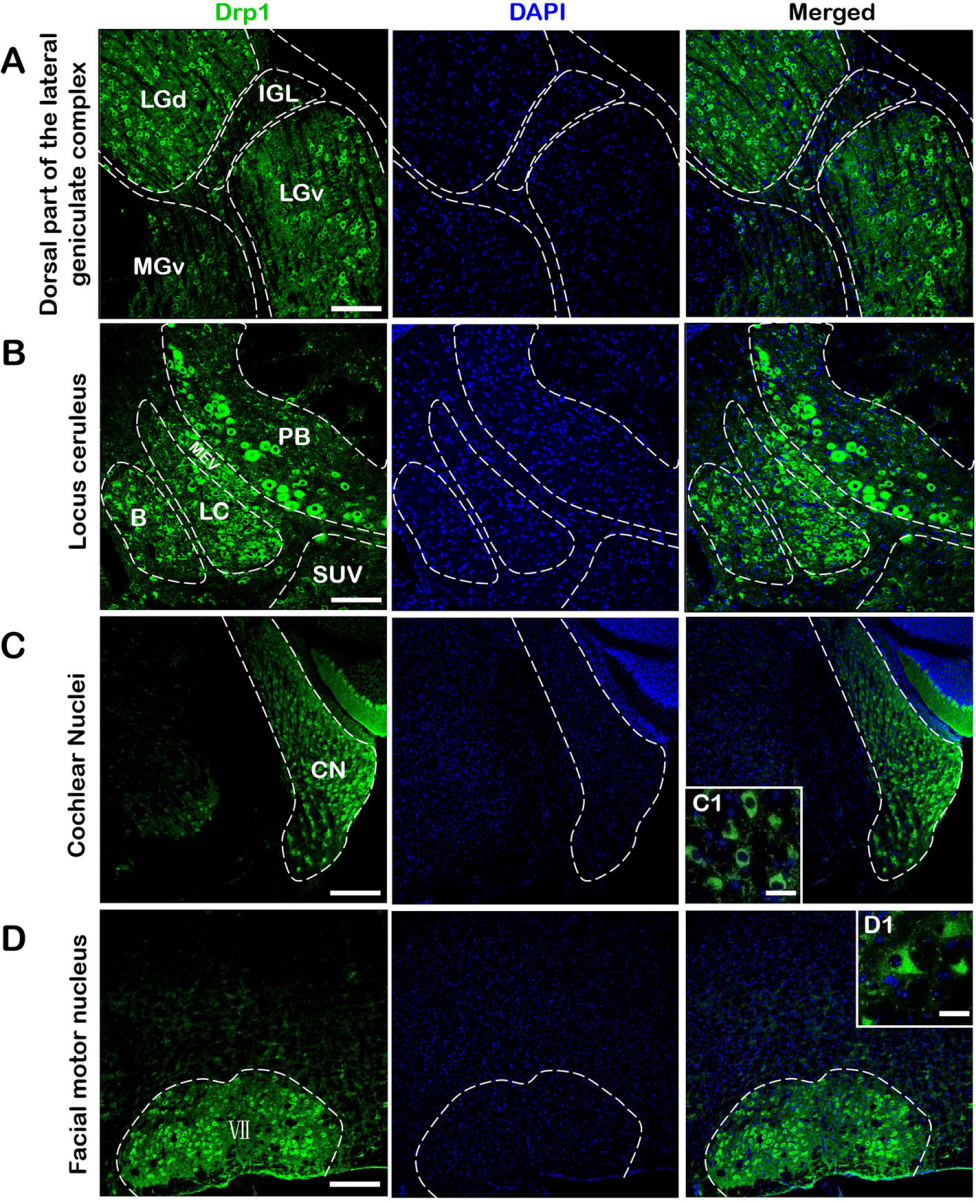
Brain regions with high Drp1 protein expression. Drp1 was highly expressed in LGd and LGv **a**, PB **b**, CN **c** and VII **d**. C1 and D1 show magnified images of C and D. Bar = 100 μm **a, b**; 200 μm **c, d**; 30 μm (C1, D1). Abbreviations: LGd: dorsal part of the lateral geniculate complex; LGv: ventral part of the lateral geniculate complex; IGL: intergeniculate leaflet of the lateral geniculate complex; MGv: medial geniculate complex, ventral part; PB: parabrachial nucleus; B: Barrington’s nucleus; LC: locus ceruleus; MEV: midbrain trigeminal nucleus; SUV: superior vestibular nucleus; CN: cochlear nuclei; VII: facial motor nucleus

Among all the regions, we selected four regions in which Drp1 was highly expressed (Fig. 3). In the thalamus, Drp1 was highly expressed in the LGd (dorsal part of the lateral geniculate complex) and LGv (ventral part of the lateral geniculate complex) (Fig. 3 a). In the pons, Drp1 expression was higher in the PB (parabrachial nucleus), LC (locus ceruleus) and B (Barrington’s nucleus) than in other nuclei (Fig. 3 b). The labeling intensity in the CN (cochlear nuclei) and VII (facial motor nucleus) achieved a strong signal (++++) (Fig. 3 c d). Details of expression of Drp1 protein in all regions are shown in Table 1. Moreover, based on the *Allen Mouse Brain Atlas* [21], we also analyzed the proportion of different Drp1 protein labeling intensities in the brain and spinal cord. The right part of the figure exhibited Drp1 protein expression (Fig. 5), with a low signal (+) accounting for the majority (59% in brain, 60% in spinal cord).

**Table 1:** Drp1 protein and mRNA expression in different brain regions and spinal cord

Abbreviation	Regions	Intensity	
		protein	mRNA
**CTX**	**cerebral cortex**		
RSPv1	retrosplenial area, ventral part, layer 1	**–**	+
RSPv2/3	retrosplenial area, ventral part, layer 2/3	**+**	++++
RSPv5	retrosplenial area, ventral part, layer 5	**++**	+++
RSPv6	retrosplenial area, ventral part, layer 6	**+**	+++
RSPd1	retrosplenial area, dorsal part, layer 1	**–**	+
RSPd2/3	retrosplenial area, dorsal part, layer 2/3	**+**	+++
RSPd5	retrosplenial area, dorsal part, layer 5	**++**	+++
RSPd6	retrosplenial area, dorsal part, layer 6	**+**	+++
PTLp1	posterior parietal association areas, layer 1	**–**	+
PTLp2/3	posterior parietal association areas, layer 2/3	**+**	+++
PTLp4	posterior parietal association areas, layer 4	**+**	+++
PTLp5	posterior parietal association areas, layer 5	**++**	+++
PTLp6	posterior parietal association areas, layer 6	**+**	+++
AUD1	auditory area, layer 1	**–**	+
AUD2/3	auditory area, layer 2/3	**+**	++++
AUD4	auditory area, layer 4	**+**	+++
AUD5	auditory area, layer 5	**++**	+++
AUD6	auditory area, layer 6	**+**	+++
TEa1	temporal association areas, layer 1	**–**	+
TEa2/3	temporal association areas, layer 2/3	**+**	++++
TEa4	temporal association areas, layer 4	**+**	+++
TEa5	temporal association areas, layer 5	**++**	+++
TEa6	temporal association areas, layer 6	**+**	+++
PIR1	piriform area, molecular layer 1	**–**	+
PIR2	piriform area, molecular layer 2	**+**	++++
PIR3	piriform area, polymorph layer 3	**+**	++
**HIP**	**hippocampal region**		
CA1so	field CA1, stratum oriens	+	+
CA1sp	field CA1, pyramidal layer	+	++++
CA1sr	field CA1, stratum radiatum	+	+
CA1slm	field CA1, stratum lacunosum-moleculare	+	++
DG-mo	molecule layer of dentate gyrus	+	+
DG-sg	granular cell layer of dentate gyrus	+	++++
DG-po	polymorphic layer of dentate gyrus	++	+++
**TH**	**thalamus**		
VPL	ventral posterolateral nucleus of the thalamus	**+**	+++
VPM	ventral posteromedial nucleus of the thalamus	**+**	+++
LGd	dorsal part of the lateral geniculate complex	**+++**	+++
IGL	intergeniculate leaflet of the lateral geniculate complex	**++**	+++
LGv	ventral part of the lateral geniculate complex	**+++**	+++
PO	posterior complex of the thalamus	**+**	+++
PF	parafascicular nucleus	N/A	+++
**MB**	**midbrain**		
PAG	periaqueductal gray	++	+++
MRN	midbrain reticular nucleus	N/A	++
IC	inferior colliculus	N/A	+++
DR	dorsal nucleus raphe	N/A	++++
**P**	**pons**		
PB	parabrachial nucleus	++++	++++
LC	locus ceruleus	+++	++++
B	Barrington’s nucleus	+++	++++
PCG	pontine central gray	+	+++
**MY**	**medulla**		
CN	cochlear nuclei	++++	+++
VCO	ventral cochlear nucleus	++++	+++
DCO	dorsal cochlear nucleus	++++	+++
CNlam	granular lamina of the cochlear nuclei	+++	+++
PARN	parvicellular reticular nucleus	+	++
VII	facial motor nucleus	++++	+++
SPVI	spinal nucleus of the trigeminal, interpolar part	+++	N/A
NTS	nucleus of solitary tract	++	N/A
ECU	external cuneate nucleus	++++	N/A
PAS	parasolitary nucleus	+	N/A
MDRN	medullary reticular nucleus	+++	N/A
SPVO	spinal nucleus of the trigeminal, oral part	N/A	++
IRN	intermediate reticular nucleus	N/A	++
sptV	spinal tract of the trigeminal nerve	N/A	+
SPIV	spinal vestibular nucleus	++	N/A
**CB**	**cerebellum**		
SIMgr	simple lobule, granular layer	–	++++
SIMpu	simple lobule, Purkinje layer	++++	++++
SIMmo	simple lobule, molecular layer	+	++
FLgr	flocculus, granular layer	–	++++
FLmo	flocculus, molecular layer	+	++
IP	interposed nucleus	++	+++
SUV	superior vestibular nucleus	N/A	N/A
MGv	medial geniculate complex, ventral part	N/A	N/A
MEV	midbrain trigeminal nucleus	N/A	N/A
**fiber tracts**			
cing	cingulum bundle	**–**	+
cc	corpus callosum	**–**	+
alv	alveus	–	+
df	dorsal fornix	–	+
fr	fasciculus retroflexus	N/A	+
scp	superior cerebelar peduncles	N/A	+
icp	inferior cerebellar peduncle	N/A	+
sctv	ventral spinocerebellar tract	N/A	+
rust	rubrospinal tract	N/A	+
**VS**	**ventricular systems**		
V4	fourth ventricle	N/A	–
V4r	lateral recess	N/A	++++
AQ	cerebral aqueduct	N/A	–
**spinal cord**			
Lamina I		+	++++
Lamina II		+	+++
Lamina III		+	+++
Lamina IV		+	+++
Lamina V		+	+++
Lamina VI		+	+++
Lamina VII		++	+++
Lamina VIII		++	+++
Lamina IX		++	+++
Lamina X		++	+++

Drp1 expression was determined based on the labeling intensity, which was classified as no signal (−), low signal (+), moderate signal (++), high signal (+++), and strong signal (++++). “N/A” represents this region was not observed or counted

In conclusion, Drp1 protein is widely distributed in the brain and spinal cord, confirming the importance of Drp1 in the CNS.

### At the mRNA level

#### Probe titer determination

Using the correctly sequenced recombinant plasmid as the template, the sequence containing the target gene fragment and SP6/T7 promoter joints at both ends was amplified. After amplification, we determined the probe concentration. The Drp1 probe carrying SP6 and T7 was 281.27 ng/μl and 202.61 ng/μl, respectively.

#### Identification of probes and antisense probes

Drp1 mRNA gene probes included the upstream and downstream promoter SP6 and T7 sequences, while the sense sequence probes served as the negative control. The results showed a high hybridization signal of SP6 probe, and specific punctate granules were observed, while the T7 probe showed no specific staining. Therefore, the antisense sequence of the target gene Drp1 with SP6 as the promoter was selected for subsequent experiments.

#### A wide distribution of Drp1 mRNA in the mouse brain and spinal cord

**Fig. 4:**
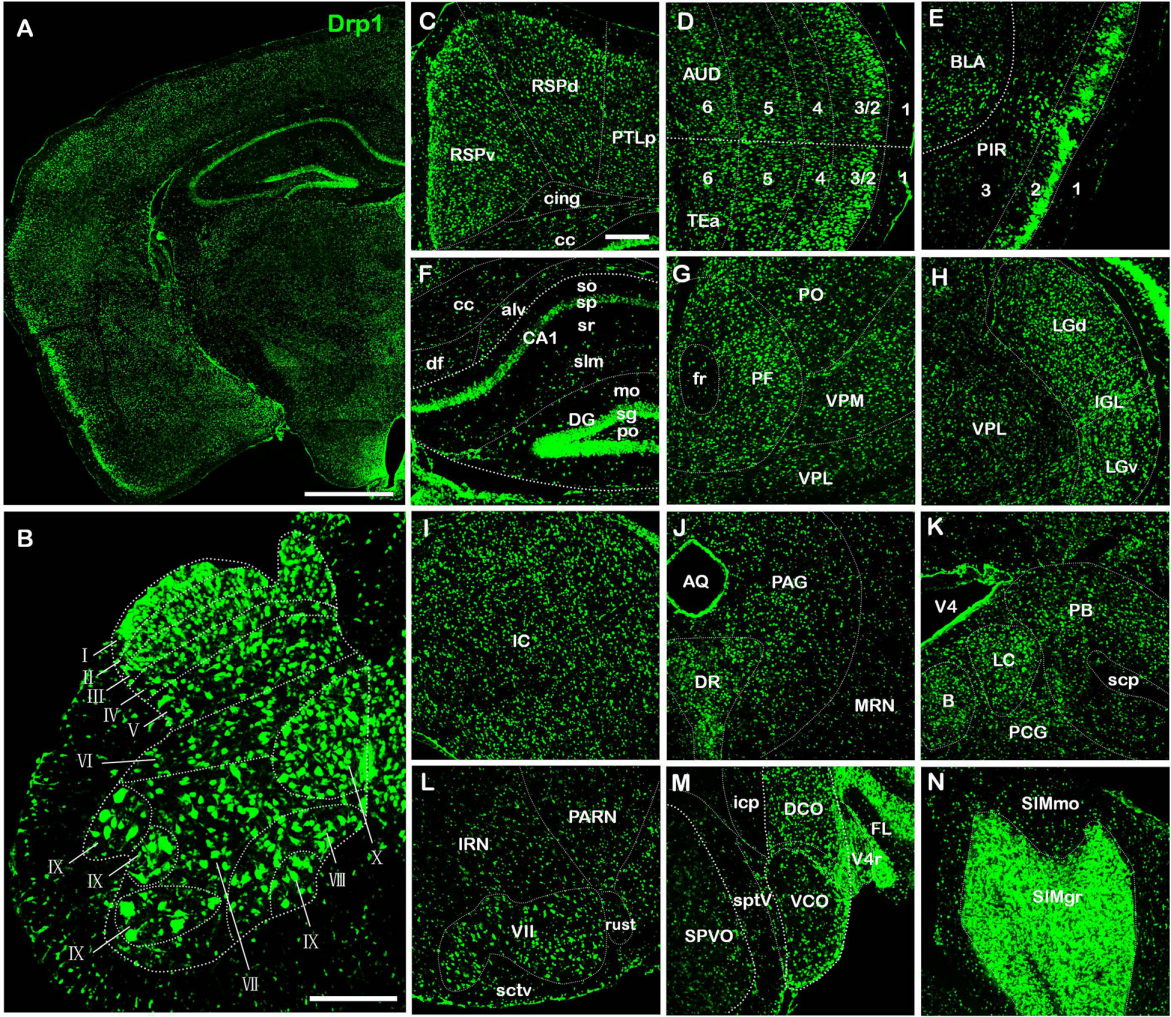
Drp1 mRNA distribution in the whole brain and spinal cord. **a**. mRNA distribution of Drp1 in the brain. The section refers to *The Allen Mouse Brain Atlas* (Mouse, P56, Coronal, image 72 of 132). **b**. mRNA distribution of Drp1 in the spinal cord. Drp1 mRNA was highly expressed in all layers (I-X). The mRNA expression of Drp1 was higher in layer I than in the other layers. **c-n**. Nuclei with high Drp1 mRNA expression. In the cerebral cortex, Drp1 was highly expressed in RSP **c**, AUD **d**, and PIR **e**. In the hippocampal region, Drp1 was highly expressed in CA1sp, DG-sg **f**. In the thalamus, Drp1 was highly expressed in PF **g**, LGv, LGd **h**, In the midbrain, Drp1 was highly expressed in IC **i**, PAG, DR **j**. In the pons, Drp1 was highly expressed in PB, LC, B, PCG **k**. In the medulla, Drp1 was highly expressed in PARN, IRN, VII **l**, VCO, DCO **m**. In the cerebellum, Drp1 was highly expressed in SIMgr **n**. Bar = 1 mm **a**; 200 μm **b-n**. Abbreviations: RSPd: retrosplenial area, dorsal part; RSPv: retrosplenial area, ventral part; PTLp: posterior parietal association areas; cc: corpus callosum; cing: cingulum bundle; AUD: auditory areas; TEa: temporal association areas; BLA: basolateral amygdalar nucleus; PIR: piriform area; alv: alveus; df: dorsal fornix; cc: corpus callosum; CA: ammom’s horn; CA1-so: field CA1, stratum oriens; CA1-sp: field CA1, pyramidal layer; CA1-sr: field CA1, stratum radiatum; CA1-slm: field CA1, stratum lacunosum-moleculare; DG-mo: molecule layer of dentate gyrus; DG-sg: granular cell layer of dentate gyrus; DG-po: polymorphic layer of dentate gyrus; PO: posterior complex of the thalamus; VPM: ventral posteromedial nucleus of the thalamus; VPL: ventral posterolateral nucleus of the thalamus; LGd: dorsal part of the lateral geniculate complex; LGv: ventral part of the lateral geniculate complex; IGL: intergeniculate leaflet of the lateral geniculate complex; fr: fasciculus retroflexus; PF: parafascicular nucleus; IC: inferior colliculus; PAG: periaqueductal gray; AQ: cerebral aqueduct; DR: dorsal nucleus raphe; MRN: midbrain reticular nucleus; V4: fourth ventricle; PB: parabrachial nucleus; B: Barrington’s nucleus; LC: locus ceruleus; PCG: pontine central gray; scp: superior cerebelar peduncles; SIMmo: simple lobule, molecular layer; SIMgr: simple lobule, granular layer; V4r: lateral recess; icp: inferior cerebellar peduncle; FL: flocculus; SPVO: spinal nucleus of the trigeminal, oral part; sptV: spinal tract of the trigeminal nerve; VCO: ventral cochlear nucleus; DCO: dorsal cochlear nucleus; IRN: intermediate reticular nucleus; PARN: parvicellular reticular nucleus; sctv: ventral spinocerebellar tract; rust: rubrospinal tract

Results showed that Drp1 mRNA was widely distributed in all mouse brain regions with a strong hybridization signal and high specificity (Fig. 4 a).

Considering all the results, four regions were selected for further description. In the thalamus, Drp1 was highly expressed in the LGd, LGv and IGL (intergeniculate leaflet of the lateral geniculate complex), with the fluorescence intensity of these three nuclei reaching a high signal (+++) (Fig. 4 h). In the pons, the expression of Drp1 mRNA reached a strong signal (++++) in the PB, LC and B. Expression was lower in the PCG (pontine central gray) than that in these nuclei (Fig. 4 k). In the medulla, Drp1 mRNA was highly expressed in the VCO (ventral cochlear nucleus) and DCO (dorsal cochlear nucleus) (Fig. 4 m). Drp1 mRNA was also widely distributed in the mouse spinal cord, demonstrating a strong hybridization signal (Fig. 4 b). Details of the Drp1 mRNA expression in other regions are shown in Table 1 and Fig. 4.

**Fig. 5:**
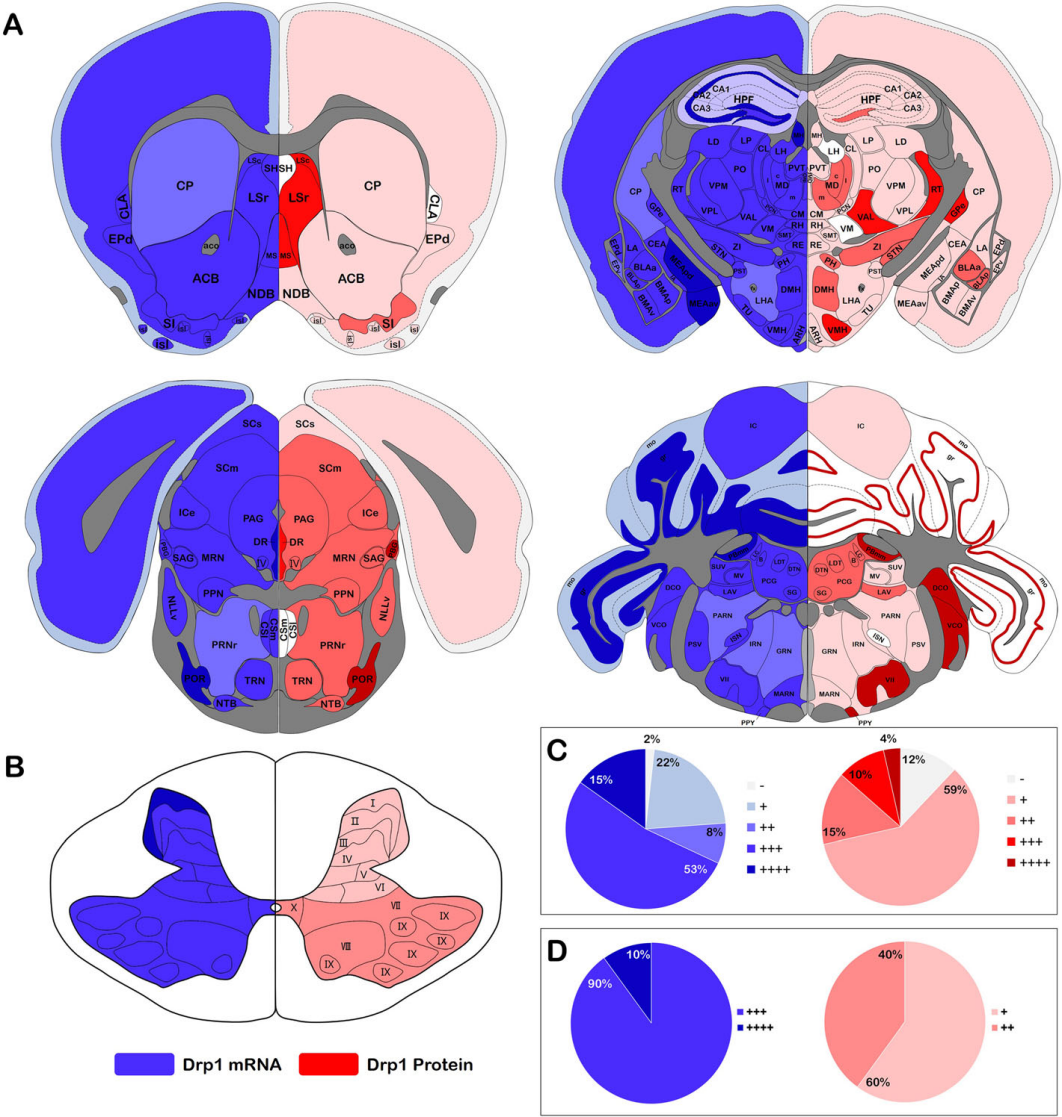
Comparison of Drp1 mRNA and protein expression. **a**. Comparison of Drp1 mRNA and protein expression in the brain. Based on the *Allen Mouse Brain Atlas*, the Drp1 signal was visualized in four different brain coronal sections. The left part shows the Drp1 mRNA expression, and the right part shows the Drp1 protein expression. **b**. Comparison of Drp1 mRNA and protein expression in the spinal cord. Drp1 mRNA was highly expressed in layer I; in the deep layers (VII, VIII, IX, X), Drp1 protein expression was slightly higher than in the superficial layers (I, II, III). **c**. Statistical analysis of the Drp1 mRNA and protein expression comparison in the brain. Drp1 expression was determined based on the labeling intensity, which was classified as no signal (−), low signal (+), moderate signal (++), high signal (+++), and strong signal (++++). The expression levels of Drp1 mRNA and protein were statistically analyzed based on 148 mice brain nuclei. The results showed that the Drp1 mRNA and protein expression levels were different in the mouse brain. In the brain, high expression of Drp1 mRNA (“+ + +” accounting for 53%) but low expression of Drp1 protein was mostly observed (“+” accounting for 59%). D. Statistical analysis of Drp1 mRNA and protein expression comparison in the spinal cord. In the spinal cord, high expression of Drp1 mRNA (“+ + +” accounting for 90%) but low expression of Drp1 protein was mostly observed (“+” accounting for 60%)

Moreover, we compared the Drp1 labeling intensity at the protein and mRNA levels. Based on the *Allen Mouse Brain Atlas* [21], the Drp1 signal was visualized in different brain slices. The left part of the figure showed Drp1 mRNA expression, and the right part showed Drp1 protein expression. The results showed that a high intensity Drp1 mRNA labeling signal (+++) accounted for the majority of the expression (53% in brain, 90% in spinal cord), which was higher than the same protein level (+++) in both the brain (10%) and spinal cord (0%) (Fig. 5).

In conclusion, Drp1 was widely distributed in the central nervous system but with heterogeneity, with some areas or nuclei showing high Drp1 expression. Heterogeneity was also found between the Drp1 mRNA and protein level.

### Mainly and highly expressed Drp1 in neurons

The localization of Drp1 in the brain and spinal cord was consistent with what has been proposed in the traditional theory that Drp1 is widely distributed in the CNS [1]. However, unequivocal proof of the innervation is still lacking.

**Fig. 6:**
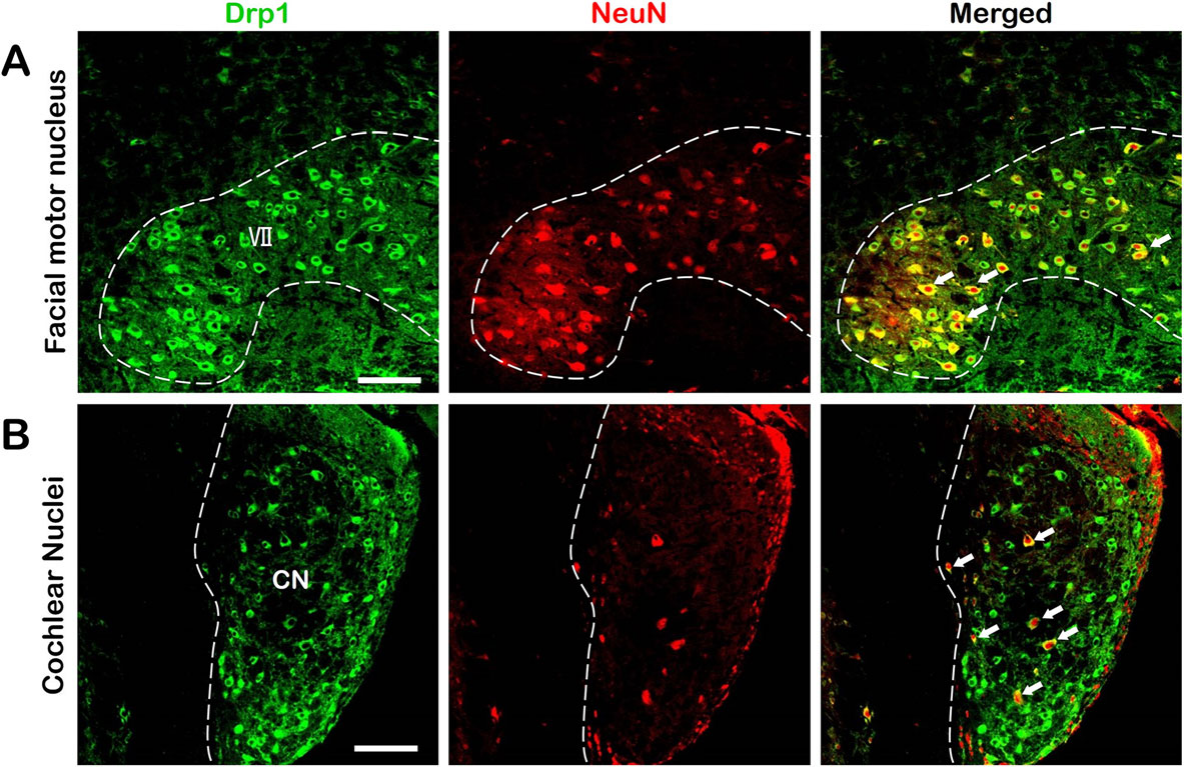
Colabeling of Drp1 and NeuN. **a**. Drp1 and NeuN staining results in VII. In this nucleus, Drp1 and NeuN were completely colabeled. **b**. Drp1 and NeuN staining results in the CN. Drp1 and NeuN were colabeled only in a few parts. In other areas of the CN expressing Drp1, however, NeuN staining was not detected. Bar = 100 μm **a, b**. Abbreviations: CN: cochlear nuclei; VII: facial motor nucleus; NeuN: neuronal nuclear antigen

To specify the Drp1 distribution in neurons, we utilized double immunofluorescence to label Drp1 and neurons with the anti-Drp1 antibody and anti-NeuN (neuronal nuclear antigen) antibody. Considering all the results, two representative nuclei were selected, and we found that the fluorescence of Drp1 (green) and NeuN (red) coincided well in the VII (Fig. 6 a). However, Drp1 and NeuN were colabeled only in a few parts of the CN, but in other areas of the CN with Drp1 expression, NeuN staining was not found (Fig. 6 b).

In conclusion, Drp1 is mainly and highly expressed in neurons in the central nervous system, and in parts of the nervous system other than neurons, low levels of Drp1 expression could also be observed.

### Highly expression of Drp1 in GABAergic neurons

**Fig. 7:**
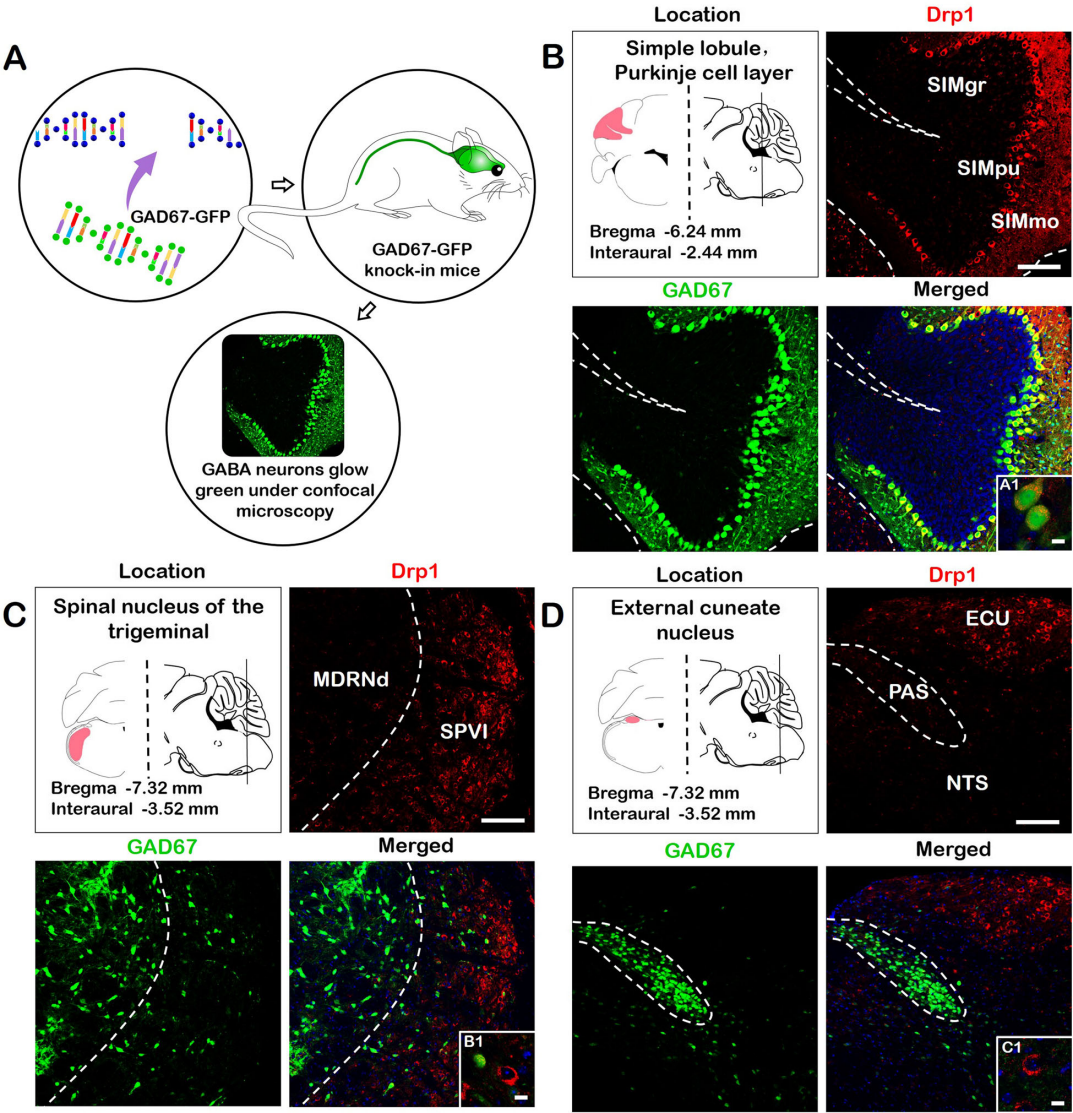
Colabeling of Drp1 and GAD67. **a**. The heterogeneous distribution of Drp1 in different kinds of neurons was further observed. GAD67-GFP transgenic mice were used to observe the colabeling of GABAergic neurons and Drp1 in different brain regions. **b**. Drp1 and GAD67 were double-labeled in SIMpu. The pu cells expressed Drp1 and GAD67, demonstrating complete colabeling. **c**. Drp1 and GAD67 in the medulla. Drp1 was highly expressed in SPVI and weakly expressed in MDRNd, while GAD67 showed lower expression in SPVI and higher expression in MDRNd. **d**. Drp1 and GAD67 in ECU. The results showed that Drp1 and GAD67 were not colabeled in most of this area. Bar = 100 μm (A-C); 25 μm (A1-C1). Abbreviations: GAD67: glutamic acid decarboxylase 67; GABA: γ-Aminobutyric acid; SIMpu: simple lobule, Purkinje layer; SIMmo: simple lobule, molecular layer; SIMgr: simple lobule, granular layer; MDRNd: medullary reticular nucleus, dorsal part; SPVI: spinal nucleus of the trigeminal, interpolar part; ECU: external cuneate nucleus; NTS: nucleus of solitary tract; PAS: parasolitary nucleus

GABAergic neurons are important inhibitory neurons, which is correlated with the development of many neurological diseases, including Huntington’s disease, Alzheimer’s disease, anxiety, panic disorder and epilepsy [22–24]. To explore the effect of Drp1 on these diseases, we further clarified the distribution of Drp1 in GABAergic neurons (Fig. 7 a). GAD67 (glutamic acid decarboxylase 67)-GFP (green fluorescent protein) transgenic mice were utilized for further observation. In GAD67-GFP transgenic mice, GABAergic neurons are specifically labeled with GFP fluorescence, which will induce spontaneous neuronal fluorescence under a confocal microscope [25–27]. Therefore, colabeling of GABAergic neurons and Drp1 in different brain regions could be observed by GFP and red fluorescence (anti-Drp1 antibody).

We selected three Drp1 and GAD67 double-stained nuclei, including SIMpu, SPVI (spinal nucleus of the trigeminal, interpolar part), and ECU (external cuneate nucleus). The results indicated that Drp1 was overexpressed in the cerebellum Purkinje layer, while Drp1 and GAD67 also colabeled well in this area. Drp1 and GAD67 colabeling was lower in other nuclei (Fig. 7).

### Drp1 was localized not only in mitochondria, but mostly in cytoplasm and dendrites

**Fig. 8:**
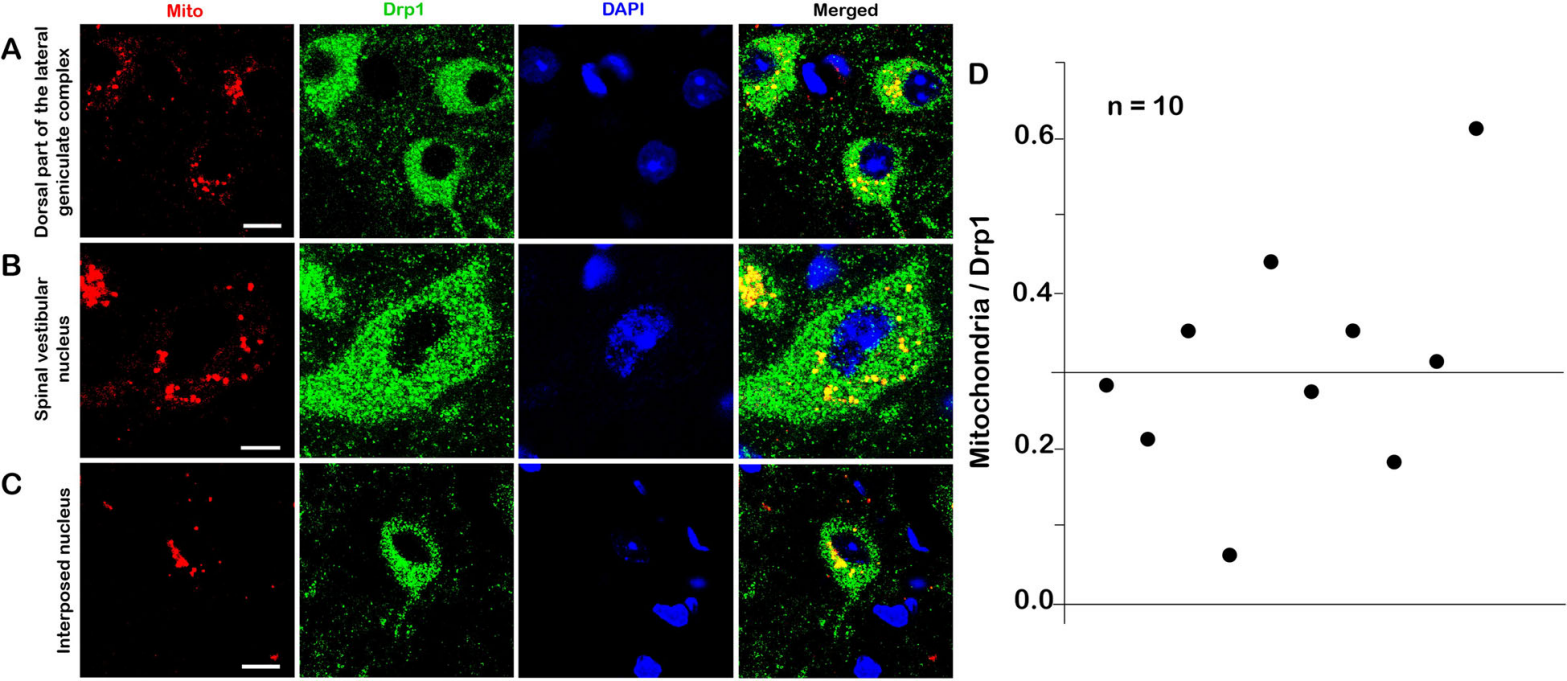
Distributions of Drp1 and Mito-Red in the brain nucleus. **a**. Drp1, Mito-Red and DAPI in the dorsal part of the LGd. Drp1 and Mito were scattered around the nucleus. **b**. Drp1, Mito and DAPI in SPIV. **c**. Drp1, Mito and DAPI in the IP. **d**. Ratio of the Mito/Drp1 fluorescence area. Ten neurons were selected for further analysis. ImageJ software was utilized to calculate the area of Mito-Red and Drp1 fluorescence in each cell. The results showed that the ratio fluctuated around 0.3. Bar = 10 μm. Abbreviations: Drp1: Dynamin-related protein 1; LGd: dorsal part of the lateral geniculate complex; SPIV: spinal vestibular nucleus; IP: interposed nucleus

**Fig. 9:**
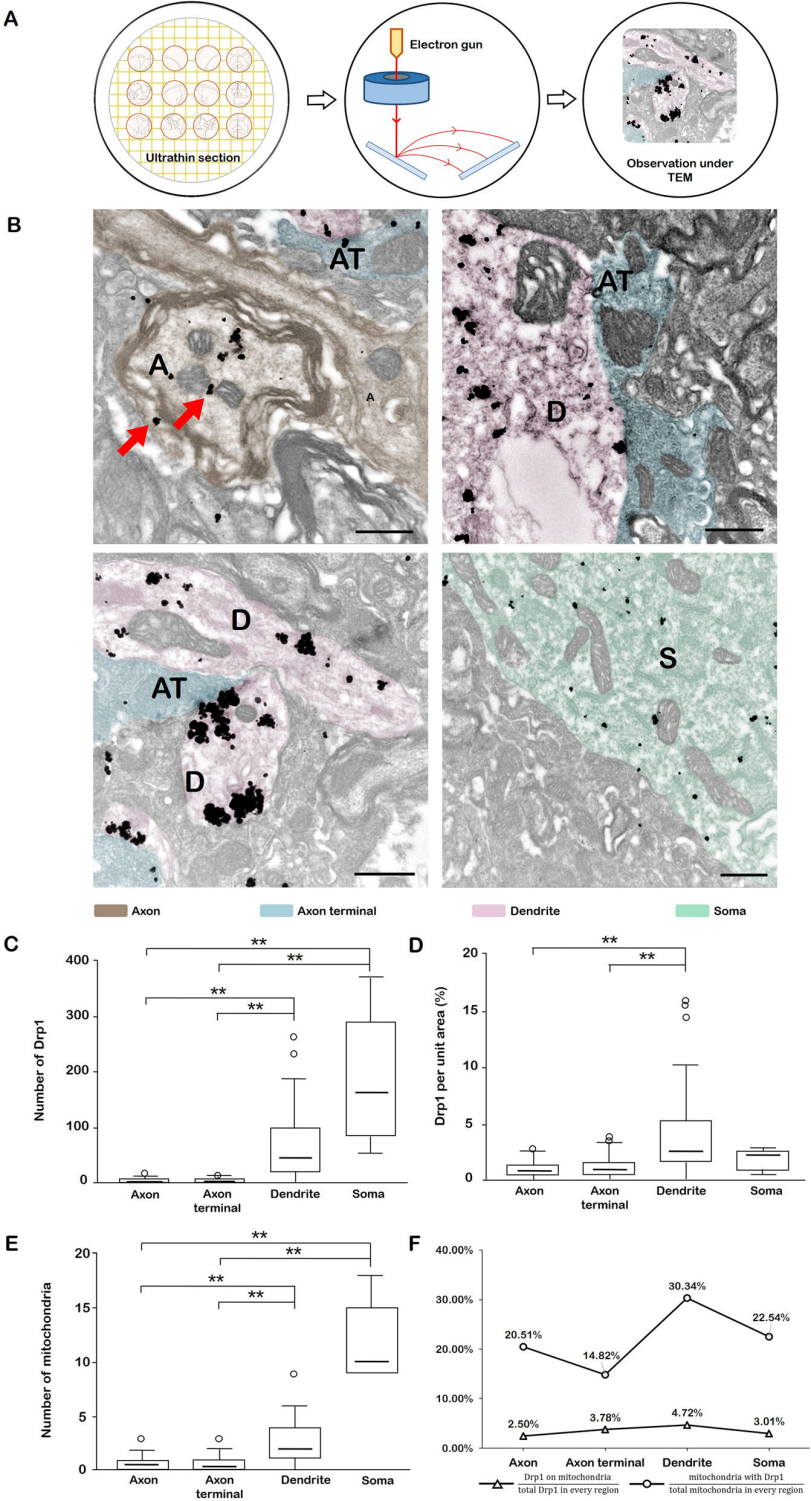
Drp1 distribution under TEM. **a**. Considering that the fluorescent particles might overlap in vertical space, the distribution of mitochondrial Drp1 in the PAG brain was further observed at the subcellular level under an electron microscope. **b**. The distribution of Drp1 under TEM. The black particulate matter is colloidal gold particles, which represent Drp1-positive expression (red arrows). Brown represents the axon (A), blue represents the axon terminal (AT), red represents dendrites (D), and green represents the soma (S). Bar =500 nm. **c**. Quantity of Drp1 in the axon, axon terminal, dendrites and soma. The Kruskal-Wallis test results showed that the quantity of Drp1 was significantly different in four regions (*p* < 0.001). The quantity of Drp1 was higher in dendrites than axons (*p* < 0.001) and axon terminals (*p* < 0.001). The quantity of Drp1 was higher in soma than axons (*p* < 0.001) and axon terminals (*p* < 0.001). **d**. The ratio of Drp1 expression per unit area. Kruskal Wallis test results showed that ratio of Drp1 expression per unit area was significantly different in four regions (*p* < 0.001). The ratio was larger in dendrites than axons (*p* < 0.001) and axon terminals (*p* < 0.001). **e**. Quantity of mitochondria in the axon, axon terminal, dendrites and soma. Using an independent-sample Kruskal-Wallis test for analysis, the results showed that the quantity of mitochondria was significantly different in four regions (*p* < 0.001). Pairwise comparisons indicated that the quantity of mitochondria was greater in dendrites than axons (*p* < 0.001) and axon terminals (*p* = 0.001). The quantity of mitochondria was greater in soma than axons (*p* < 0.001) and axon terminals (*p* < 0.001). **f**. Proportion of Drp1 in mitochondria/total Drp1 in every region and mitochondria with Drp1/total mitochondria in every region. The proportion of Drp1 in mitochondria/total Drp1 in every region was quantified. The results showed that in dendrites, 4.72% of the Drp1 was localized to mitochondria (maximum), while in axons this proportion was only 2.5% (minimum). Similarly, the proportion of mitochondria with Drp1/total mitochondria in every region was also calculated. The results showed that in dendrites, 30.34% of the mitochondria localized with Drp1 (maximum), and this was proportion only 14.82% in axonal terminals (minimum). * *p* < 0.05, ** *p* < 0.01. Abbreviations: TEM: transmission electron microscope; PAG: periaqueductal gray

As we discussed above, the relationship of Drp1 and mitochondria to many neurologic diseases has been confirmed [2]. Moreover, Drp1 is significant in maintaining mitochondrial function through the balanced control of mitochondrial fission and fusion [8]. Therefore, we observed the localization of mitochondrial Drp1 in the brain nucleus. Double labeling of Drp1 (green) and Mito-Red (red) in the LGd, SPIV (spinal vestibular nucleus) and IP (interposed nucleus) showed that Mito-Red completely colocalized with Drp1, while in contrast, the range of the Drp1 distribution was wider than the Mito-Red. Both Drp1 and Mito-Red were scattered around the nucleus. Mito-Red was concentrated on one side of the cell nucleus, while Drp1 expression showed no polarity and connected to form a network structure around the nucleus. In conclusion, the size of the green (Drp1) granules was smaller than the red (Mito-Red) one, but the distribution range of Drp1 was wider than Mito-Red, indicating that Drp1 was localized not only to mitochondria, but mostly in other regions (Fig. 8). Considering that the fluorescent particles may overlap in vertical space, we utilized an electron microscope for further observations (Fig. 9 a).

Next, we investigated the distribution of mitochondrial Drp1 in the PAG (periaqueductal gray) at the subcellular level under an electron microscope. The immune colloidal gold technique was utilized to label Drp1. Colloidal gold was the tracer, which could combine with the positively charged protein molecules in the weak alkaline environment [28, 29]. In Fig. 9, the black particulate matter is colloidal gold particles, which represent positive Drp1 expression (red arrows). The results showed that Drp1 was mainly expressed in the cytoplasmic matrix, which was consistent with our above results. Dendrites could also exhibit some expression, and a small amount of punctate Drp1 expression was distributed on the mitochondrial membrane (Fig. 9 b).

We then further investigated the quantity of mitochondria and Drp1 in axons, axon terminals, dendrites and soma. Seventy axons, 37 axon terminals, 36 dendrites and 6 somas were counted, and the average quantity of mitochondria and Drp1 in each part was calculated. Utilizing the Kruskal-Wallis test, we analyzed Drp1 expression and its correlation with mitochondria from four aspects (Fig. 9 c-f):

A) Quantity of Drp1

The quantity of Drp1 was significantly different in four regions (*p* < 0.001). The quantity of Drp1 was greater in dendrites than axons (*p* < 0.001) and axon terminals (*p* < 0.001). The quantity of Drp1 was greater in soma than axons (*p* < 0.001) and axon terminals (*p* < 0.001)

B) Ratio of Drp1 expression per unit area

The ratio of Drp1 expression per unit area was significantly different in four regions (*p* < 0.001). The ratio was larger in dendrites than axons (*p*<0.001) and axon terminals (*p*<0.001).

C) Quantity of mitochondria

The results indicated that the quantity of mitochondria was significantly different in four regions (*p* < 0.001). The quantity of mitochondria was greater in dendrites than axons (*p* < 0.001) and axon terminals (*p* = 0.001). The quantity of mitochondria was greater in soma than axons (*p* < 0.001) and axon terminals (*p* < 0.001).

D) Proportion of Drp1 on mitochondria/total Drp1 in every region and mitochondria with Drp1/total mitochondria in every region

The proportion of Drp1 on mitochondria/total Drp1 in every region was counted. The results showed that in dendrites, 4.72% of the Drp1 was localized to mitochondria (maximum), while in axons this proportion was only 2.5% (minimum).

Similarly, the proportion of mitochondria with Drp1/total mitochondria in every region was also calculated. The results showed that in dendrites, 30.34% mitochondria were localized to Drp1 (maximum), and this proportion was only 14.82% in axonal terminals (minimum). *p < 0.05, **p < 0.01.

### Drp1 expression in human malignant glioma tissue reached the highest value in grade III and then declined

As stated above, in components other than neurons, Fig. 6 also showed a low level of Drp1 expression. Glial cells represent another kind of vital component in the brain other than neurons, and some previous research has demonstrated a relationship between Drp1 and glial cells. In models of ischemic injury, Zhou et al. demonstrated an upregulation of Drp1 and enhanced mitochondrial fission in microglia [30]. Chae et al. revealed that Drp1-mediated mitochondrial fission could activate microglial cells by regulating p62-mediated autophagy [31]. Considering the crucial role of Drp1 in glial cells, we speculated that the other regions expressing Drp1 were probably glial cells.

Therefore, we also analyzed Drp1 expression in human malignant glioma tissue. With the normal human brain as the control tissue, grade I represented juvenile glioma tissue and grade II-IV represented glioma tissue with mild, moderate and severe malignancy, respectively.

**Fig. 10:**
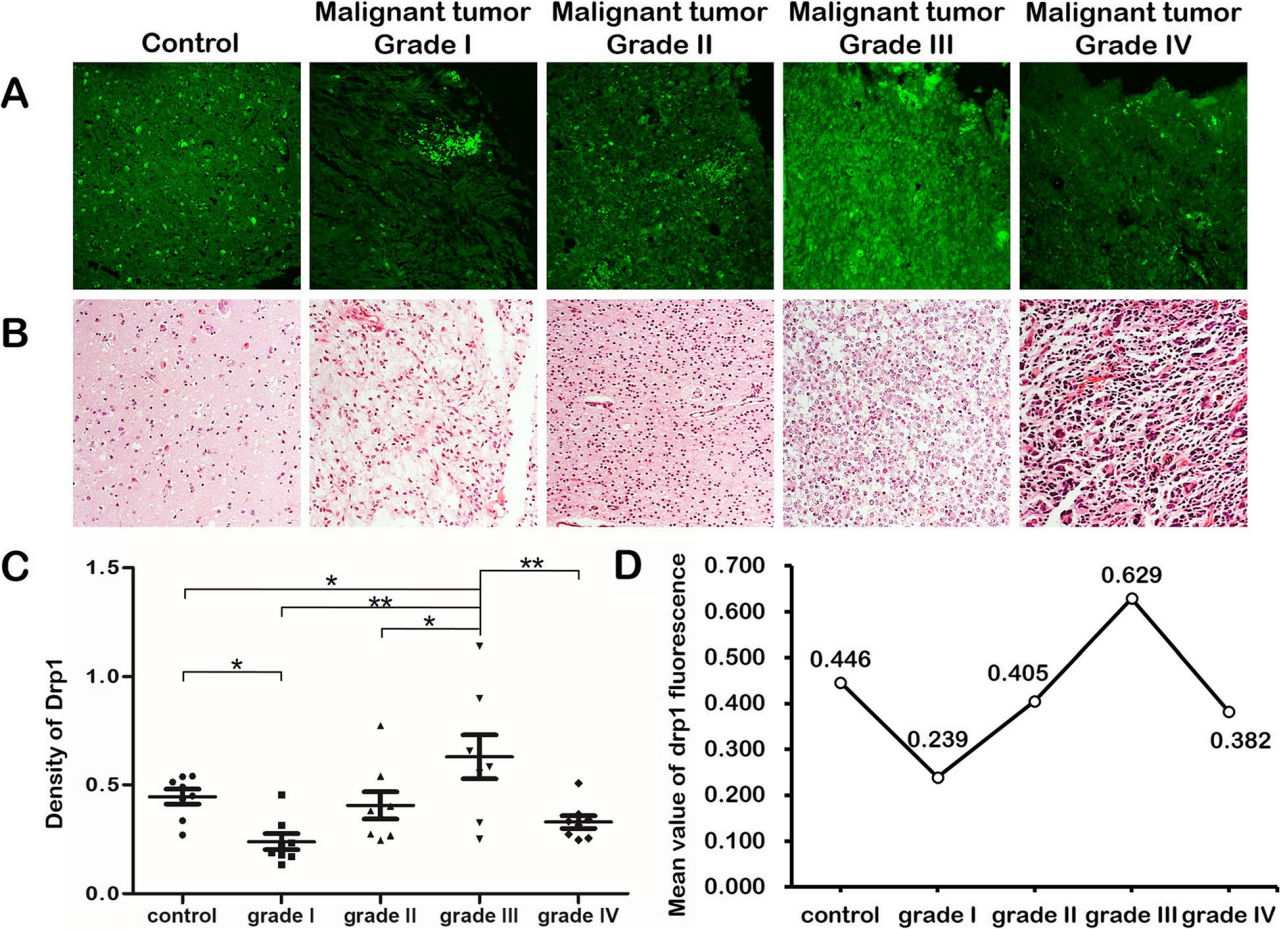
Drp1 expression in human malignant glioma tissue. **a, b**. Drp1 immunofluorescence staining and HE staining of the same area in control tissue and human malignant glioma tissue grade I-IV. **c**. Comparison of the Drp1 fluorescence intensity in tissues of different grades. Eight fluorescent spots were randomly selected for every grade. The Drp1 fluorescence intensity was calculated using ImageJ software. One-way ANOVA was used for statistical analysis. The results showed that Drp1 expression was significantly different in glioma I-IV grade (*p* = 0.01). After the LSD test, Drp1 expression was higher in the control group than grade I (*p* = 0.018). In contrast, it was higher in grade III than the control group (*p* = 0.036), grade I (*p* < 0.001), grade II (*p* = 0.011), and grade IV (*p* = 0.001). **d**. The mean Drp1 fluorescence intensity was calculated for every grade. From grade I-III, the mean Drp1 fluorescence intensity showed an increasing trend. In grade IV, the mean Drp1 fluorescence intensity was significantly reduced. * *p* < 0.05, ** *p* < 0.01. Abbreviations: LSD: least significant difference

Eight fluorescent spots were randomly selected in every tissue grade. The Drp1 fluorescence intensity was calculated using ImageJ software (Table 2 & Fig. 10). One-way ANOVA was used for the statistical analysis. The results revealed significantly different Drp1 expression in glioma grade I-IV (*p* = 0.01). After the LSD (least significant difference) test, Drp1 expression was higher in the control group than grade I (*p* = 0.018). In contrast, it was higher in grade III than the control group (*p* = 0.036), grade I (*p* < 0.001), grade II (*p* = 0.011), and grade IV (*p* = 0.001). * *p* < 0.05, ** *p* < 0.01.

**Table 2:** Value of Drp1 fluorescence intensity in different glioma grades

	Control	grade I	grade II	grade III	grade IV
**1**	0.49	0.134	0.273	0.326	0.254
**2**	0.433	0.232	0.362	0.618	0.273
**3**	0.538	0.454	0.267	0.9	0.364
**4**	0.54	0.314	0.539	0.556	0.508
**5**	0.45	0.18	0.773	0.585	0.67
**6**	0.269	0.19	0.245	0.252	0.332
**7**	0.513	0.173	0.381	0.657	0.339
**8**	0.335	0.234	0.402	1.139	0.316
**Mean value**	0.446	0.239	0.405	0.629	0.382

Eight fluorescent spots were randomly selected in every grade. Value of Drp1 fluorescence intensity was calculated by ImageJ software. One-way ANOVA was used for statistical analysis Results showed that Drp1 expression was significantly different in glioma I-IV grade (*p* = 0.01). After LSD test, Drp1 expression in control group was higher than that in grade I (*p* = 0.018). On the contrary, grade III was higher than that in control group (*p* = 0.036), grade I (*p* < 0.001), grade II (*p* = 0.011), and grade IV (*p* = 0.001)

In conclusion, from grade I-III, the mean Drp1 fluorescence intensity showed an increasing trend. The mean Drp1 fluorescence intensity was significantly reduced in grade IV (Fig. 10 c, d).

## Discussion

The present study revealed a wide distribution of Drp1 in the central nervous system at both the protein and mRNA level, confirming the importance of Drp1. Moreover, Drp1 was widely expressed in the cytoplasm but barely in mitochondria, which is consistent with previous studies [1, 2]. However, the distribution of Drp1 also showed heterogeneity. Based on the heterogeneous distribution of Drp1, we discuss four possibilities underlying this phenomenon.

### The heterogeneous distribution of Drp1 in dendrites provides the basic requirements for dendrite formation

All the immunofluorescence results showed the wide expression and distribution of Drp1 around the cell nucleus. In addition, we found that Drp1-positive protuberant structures (long strips in Fig. 2 b) were potentially axons or dendrites. The IEM (immunoelectron microscopy) results further revealed that Drp1 was mainly expressed in the cytoplasmic matrix, while dendrites also showed some expression. Kruskal-Wallis analysis confirmed the heterogeneous distribution of Drp1 in different neuronal compartments. Together with the IEM and Kruskal-Wallis analysis results, we concluded that the amount of Drp1 was greater in the soma and dendrites than axons and axon terminals. Interestingly, the number of mitochondria showed the same characteristics. Moreover, the highest ratio of dendrites (Drp1 in mitochondria/total Drp1 in every region, mitochondria with Drp1/total mitochondria in every region) also indicated that Drp1 and its related mitochondrial dynamics played a vital role in this compartment.

The heterogeneous distribution of Drp1 in dendrites was probably indicative of its role in dendrite formation. By utilizing shRNAs to knockout Drp1, Itoh et al. revealed the crucial role of Drp1 in controlling primary dendrite formation in neurons. First, they demonstrated an enrichment of Drp1 in postsynaptic terminals (the majority is dendrite), which was consistent with our research. Second, in cultured neurons, they found that the loss of Drp1 induced ectopic dendrite extension, further affecting brain function [32]. In addition, during the process of controlling dendrite formation, Drp1 was correlated with the mitochondrial distribution. Using molecular manipulations to inhibit Drp1 expression, Li and colleagues observed a decline in dendritic mitochondria, leading to the loss of dendritic spines [33]. Moreover, in a mutant Drp1 fruit fly model, Verstreken et al. also revealed the absence of mitochondria in neuron postsynaptic terminals [34]. Our electron microscopy calculations showed that the quantity of mitochondria was significantly different in four regions, with a larger amount in in dendrites than other compartments, confirming the abovementioned correlation between dendritic Drp1 and mitochondria. Therefore, the heterogeneous distribution of Drp1 in dendrites further indicated its vital role in normal neuronal function, and provided the basic requirement for dendrite formation.

In contrast, compared with dendrites, axons and axon terminals showed a lower Drp1 expression level. Considering that synaptic vesicle trafficking leads to the largest metabolic burden in presynaptic terminals, and this huge energy demand is pivotal in neuronal transmission [35], we provide two possible speculations. First, our experimental samples and data must be further analyzed. Second, neurons might contain some other molecules that could also induce the mitochondrial fission process in other neuronal compartments. Although many proteins participate in the mitochondrial fission mechanism, including Fis1 (fission protein 1), Mff (mitochondria fission factor) and MiD49, MiD51 (mitochondria dynamics proteins of 49 and 51 kDa), Drp1 is still known as the crucial factor [36]. Therefore, further investigation is still needed to reveal other potential fission factors.

Moreover, the difference in Drp1 protein and mRNA expression has further confirmed this speculation. As the vital factor in regulating the mitochondrial fission process, Drp1 should be expressed in all cells. However, as shown in Fig. 5, Drp1 protein and mRNA expression differ in the same region, and no Drp1 expression was detected in some neurons, which might suggest that some other molecules could also regulate mitochondrial dynamics. Further observations are still needed.

### The heterogeneous distribution of Drp1 may contribute to the occurrence and development of neurological diseases

As we discussed above, mitochondrial dysfunction is involved in the occurrence and development of neurologic disease [37]. Drp1 mutation promotes mitochondrial dysfunction [38]. A large number of studies have also demonstrated that GABA is related to the development of neurological diseases.

GABA is the major inhibitory neurotransmitter in the CNS. It has been confirmed that dysfunction of GABA metabolism or GABAergic inhibitory neurons is associated with many neurological diseases, including Huntington’s disease, Alzheimer’s disease, anxiety, panic disorder and epilepsy [23, 24]. However, whether Drp1 directly affects these diseases through GABAergic inhibitory neurons is still unclear.

In the process of mapping the Drp1 distribution in the brain and spinal cord, we found a heterogeneous expression pattern of Drp1 in some inhibitory neurons. Figure 2.B shows that Drp1 was expressed in neurites of the mo layer in the cerebellar cortex. The neurons in this layer have important inhibitory effects [39]. In addition, Drp1 is also expressed in the second layer of the cerebral cortex, which is the external granular layer [40]. The difference in Drp1 protein expression in the cerebral cortex may be related to the diverse cell types in each layer. The second layer contains a large number of granular cells, the majority of which are GABAergic inhibitory neurons [41].

Therefore, we further analyzed Drp1 expression in GABAergic neurons by utilizing GAD67-GFP knock-in mice. The results showed that the Drp1 distribution was concentrated in GABAergic inhibitory neurons. This heterogeneous Drp1 distribution among different kinds of neurons may contribute to the occurrence and development of neurological diseases.

As we discussed in the Introduction, some molecules have been found that target Drp1, including P110, mdivi-1 and OND. However, the impact of these molecules on the human body and their potential use in the clinic requires further study. Therefore, the heterogeneous distribution of Drp1 in GABAergic neurons may allow targeted treatment guidance for GABA-related diseases.

### Drp1 expression in human malignant glioma further demonstrates the significance of Drp1 in cells

Based on the above results, we concluded that Drp1 was widely but heterogeneously distributed in the central nervous system, which firmly supported the importance of this molecule. However, in the central nervous system, glial cells also contribute to the regulation of normal brain function. In Fig. 6, together with neurons, we observed low levels of Drp1 expression that other part of the nervous system. As stated above, we speculated that the other regions expressing Drp1 were likely to be glial cells.

Therefore, we further analyzed Drp1 expression in human malignant glioma tissue. Compared with the mean Drp1 fluorescence intensity in every grade, we observed an increasing Drp1 fluorescence intensity trend from grade I-III. For grade IV, the mean Drp1 fluorescence intensity was significantly reduced. The highest fluorescence intensity in the moderate malignancy indicated that the changes in mitochondrial dynamics had reached a maximum. During the process of glioma growth, some cells were found to be far from the vasculature and lacked nutrients. Under this circumstance of energetic stress, Drp1 upregulation could benefit the maintenance of the stem cell population and activity [18]. In grade IV, most of the normal tissue was destroyed, so the fluorescence intensity was significantly reduced. Such changes in expression have also been observed in other malignant tumors [42, 43].

Thus, the expression of Drp1 in human malignant glioma further demonstrated its significance in cells, and it might provide guidance that changes in Drp1 could serve as an early indicator of malignant diseases.

### Inhibition of Drp1 represent a novel target for neurological diseases treatment

When the mitochondrion is going to divide, Drp1 will translocate from the cytoplasm to the mitochondrial outer membrane with the help of receptors and adapters [44], and assemble into ring-like structures on the mitochondrial outer membrane, leading to mitochondrial fission [45, 46]. When Drp1 is translocated abnormally to the mitochondrial membrane, mitochondrial dynamic homeostasis regulated by Drp1 will be disrupted, leading to changes in mitochondrial morphology [17]. Therefore, inhibiting the Drp1 harbored in neurons under pathological state may represent a novel target in the treatment of neurological diseases [47].

For treatment, we summarize three molecules that can inhibit Drp1. P110 can effectively inhibit the translocation of Drp1 from the cytoplasm to mitochondria, and inhibit the binding of Drp1 to Fis1, thus inhibiting mitochondrial division [17]. Kanda et al. observed a significant increase in Drp1 in the neuropathic pain model. Further study demonstrated that intrathecal Drp1 antisense ODN (oligodeoxynucleotide) could decrease spinal Drp1 expression [48]. Additionally, the mdivi-1 is another Drp1 inhibitor. Luiz F also revealed that mdivi-1 could attenuate the neuron pathologic changes [16].

There is currently still no superior method for the prevention of neurological diseases, such as AD, HD and glioma. Additionally, diagnosis of the disease often lags behind its development. Therefore, the development of new target drugs is urgently needed. It is clear that P110, mdivi-1 [49, 50] and OND can effectively inhibit Drp1 translocation from the cytoplasm to mitochondria. However, the impact of the abovementioned molecules on the human body and whether they can be efficiently and conveniently applied in the clinic require further study.

The present study can be improved in terms of several features. It is much better to use Drp1 knockout mice in investigations of the distribution of Drp1. Moreover, the phosphorylation status of Drp1 is essential for its interaction with Mff, and it plays a vital role in regulating mitochondrial dynamics; however, the phosphorylation status of Drp1 was not observed herein. We will examine this phenomenon in our subsequent research.

In the present study, we observed the distribution of Drp1 in the mouse CNS and found that it was widely, yet heterogeneously, distributed in the central nervous system. The heterogeneous distribution of Drp1 may be involved in the occurrence and development of neurological diseases. Moreover, we summarized three targeted molecules for treatment. We hope that this research on the relationship between Drp1 and mitochondria in neurons may facilitate molecular therapy and provide clinical guidance for neurological disease treatment. As the vital factor in mitochondrial dynamics, future studies are still needed to examine the distribution and translocation of Drp1 beyond the pathological changes.

## Materials and methods

### Animals

Adult male C57BL/6 mice and GAD67-GFP knock-in mice (Center of Lab Animals, Fourth Military Medical University, Xi’an, China), weighed 25–30 g. In GAD67-GFP transgenic mice, GABAergic neurons are specifically labeled with GFP fluorescence, which will emit neurons spontaneous fluorescence under the confocal microscope. The generation and characterization of the GAD67-GFP knock-in mice has been described in our previous research [25, 27, 51].

Experimental mice were housed and treated in strict accordance with the Rules for Animal Care [52] and Use for Research and Education of Fourth Military Medical University.

### Experimental procedure

#### Mouse stereotaxic brain atlas

Brain figures were checked with *The Allen Mouse Brain Atlas* [21, 53] and *The Mouse Brain in Stereotaxic Coordinates, 2nd ed* [54]. Spinal cord figures were checked with *Neuroanatomy: An Illustrated Colour Text, 5th ed* [55]. All figures were prepared using Adobe Photoshop 7.0.

#### Intrathecal Mito-red loading

In order to explore the exact Drp1 distribution on mitochondria, intrathecal Mito-Red loading was utilized to visualize mitochondrial morphology.

The characterization of MitoTracker® has been described in our previous research [15]. Mito-Red (MitoTracker® Deep Red FM, Invitrogen, USA) was injected intrathecally. Mito-Red was qualified to concentration of 100 nM dissolving in 1:1 mixture of DMSO (dimethylsulfoxide). 5 μl solution was injected into subarchnoid space from a small hole on L3 vertebral lamina using a Hamilton syringe attached to 10-gauge needle. Control groups were injected with 5 μl saline using the same methods.

After 4 days, mice were perfused with 0.01 M phosphate-buffered saline (PBS; pH 7.4) and 4% formaldehyde in PBS successively after anesthesia. Brains were removed into 30% sucrose solution for tissue dehydration. After dehydration, brains were sliced into 30um using freezing microtome (CM1950, Leica). Incubation of Drp1 and fluorescence developing were same as immunofluorescence staining.

#### Immunofluorescence staining

In order to map the Drp1 protein expression in central nervous system, immunofluorescence staining was utilized in three kinds of animals, including normal adult C57BL/6 mice, intrathecal injection of Mito-Red mice and GAD67- GFP mice. Light should be avoided during Mito-Red mice operation, other operations were same to C57BL/6 mice and GAD67- GFP mice. Mito-Red mice and GAD67- GFP mice will emit spontaneous fluorescence under confocal microscope. Therefore, slices of these two groups only need single label of anti-Drp1 antibody incubation.

Mice were perfused with 0.01 M PBS and 4% formaldehyde in PBS successively after anesthesia. Brains and spinal cord were removed into 30% sucrose solution for tissue dehydration. After dehydration, brains were sliced into 30um using freezing microtome (CM1950, Leica, Germany). To determine the distribution and localization of Drp1, slices were transferred to PBS and then 10% calf serum for 30 min. Then slices were incubated with 1:250 diluted anti-Drp1antibody (ab184247, Abcam, UK) at 4 °C overnight. In order to avoid false positive results, Tempol (ROS scavenger) was utilized to treat the tissue. After anti-Drp1antibody incubation, the tissue was treated in Tempol (380 nmol/5 μ L) solution for 1 h. Then slices were incubated with 1:500 diluted secondary antibody (goat anti-rabbit IgG; Sigma, St. Louis, MO, USA) for 2 h. Slides were rinsed triple times for 15 min with 0.01 M PBS after incubation. No signal was detected when the primary or secondary antibody was omitted. Images were recorded using confocal microscopy (FV1000, Olympus, Japan) connected to an inverted microscope.

#### FISH (fluorescence in situ hybridization)

In order to observe the Drp1 mRNA distribution in central nervous system, fluorescence in situ hybridization was utilized. Drp1 gene primer was designed and used in our previous research [56].

Drp1 gene upstream primer 5′-3′: GCTCAGTGCTGGAAAGCCTA; downstream primer 5′-3′: GATGGATTGGCTCAGGGCTT, amplification length: 297 bp.

Construction of probe plasmids: cDNA of C57BL/6 mice were amplified by PCR with primers; gel was used to recycle the PCR products; the products were linked to carriers at room temperature with T7 and SP6 promoters at both ends (Roche, Switzerland); the plasmids were transformed into E.coli DH5alpha and cultured; the probe plasmids were extracted by Plasmid Extraction Kit (Tiangen, China) and then were sequenced.

Probe preparation: using the sequenced plasmid as template, PCR amplification was carried out with T7 and SP6 specific primers, PCR products were recycled by Omega Gel Recycle Kit (Omega, USA), probe was transcribed into cRNA in vitro by T7-RNA polymerase/ SP6-RNA polymerase.

Mice were anaesthetized with 25% uratan solution (6 ml/kg, intraperitoneal injection), rinsed the blood from right atrial appendage with 30 ml (0.01 M DEPC-PBS), then rinsed the blood with 4% paraformaldehyde 100 ml. After perfusion, the mice brain and lumbar spinal cord (lumbar enlargement) were removed completely, and were put in 4% paraformaldehyde fixative solution in the refrigerator at 4 °C for 24 h. Then slices were removed in 30% DEPC sucrose solution in 4 °C refrigerator for 48 h. Coronal sections were cut by Leica CM1950 (Germany). Brain slices and spinal cord slices were 30 and 25um thick, respectively. Slices were treated in 0.1 M DEPC-PB containing 2% H2O2 for 10 min, 0.1 M DEPC-PB containing 0.3% Triton X-100 for 20 min and acetylation solution for 10 min at room temperature, respectively. Slices were incubated in hybridization buffer at 58 °C for 1 h.Drp1 cRNA probe was added to the above-mentioned hybridization buffer (final concentration: 1 μg/ml) and incubated in the hybridization oven at 58 °C for 20–24 h. Rinsed slices and treated it with RNA enzyme solution for 5 min at room temperature, 2 * SSC and 0.2 x SSC (diluted by 20 * SSC, with 2% NLS) was used to rinse the slices twice, 20 min each time, 37 °C, respectively. Slices were incubated with antibody POD-anti-DIG (1:1500) for whole night, β-D-Glucose (1:100) for 30 min, FITC-avindin (1:500), for 3 h and DAPI (1:1000) for 15 min at room temperature. After incubation, the slices were observed and photographed by laser confocal microscopy (FV1000, Olympus, Japan).

#### Western blotting and analysis

In order to preliminarily explore whether the Drp1 protein has regional specific distribution characteristics, western blot was used to detect the protein in mice central nervous system.

10% SDS–polyacry-lamide gel electrophoresis (SDS-PAGE) was prepared [57]. Equal amounts of protein (30 μg) were electrophoresed on 10% SDS–polyacrylamide gels and transferred 2 h onto PVDF-membrane, which were incubated with anti-Drp1 antibody (1:1500, Abcam, UK) and secondary antibody (horse-radish peroxidase-conjugated anti-rabbit IgG from donkey, Amersham, Biosciences, Piscataway, NJ, USA) [57]. Immunoblots were developed using ECL kit (K-12045-D10; Advansta, USA) and BIO-RAD ChemiDoc MP lighting machine, results were quantified by computerized scanning densitometry and analyzed by Image J software.

#### IEM (Immunoelectron microscopy)

In order to explore Drp1 localization on mitochondria in subcellular level, we utilized immunoelectron microscopy for further observation. IEM contains different labeling methods. In this experiment, immune colloidal gold technique was utilized to label Drp1. The colloidal gold was tracer, and could be polymerized into gold particles of specific size under the action of reducing agent, and further combined with positively charged protein molecules in the weak alkali environment [28, 29].

The shooting area was PAG. After perfusion, the brain was removed immediately and was cut into small pieces (PAG area). The tissue was fixed with 4% paraformaldehyde fixative of 15% saturated picric acid, and then was sliced into 50 um thick. Then slices were used vibration microtome (DTK-1000, D.S.K., Japan) to obtain coronary section. And slices were treated into frozen section by liquid nitrogen, and were incubated with 20% donkey serum (diluted by 0.05 M TBS) for 30 min, rabbit anti-Drp1 antibody (1:100, Abcam, UK), 2% donkey blood were added afterwards for 24 h’ incubation. After primary incubation, sheep anti-rabbit (1:100, Nanoprobes, USA) labeled with immune NG (nanogold particles) and 2% donkey serum (0.05 M TBS diluted) were added for further incubation overnight at room temperature. Silver-enhanced reaction: HQ Sliver Kit (Nanoprobes, USA) treatment for 7–14 min, then dripped into Ainitator, B moherntor, C activator reagent one drop, respectively. After 7 min reaction, the slices were poured into distilled water to stop the reaction. Then, slices were incubated with 1% osmic acid solution for 35 min, then treated 70% alcohol and 1% uranyl acetate for 40 min. Then slices were removed into 70% ~ 100% gradient alcohol and propylene oxide for dehydration. Slices were polymerized in the embedding agent overnight. After stained with lead citrate, slices could be observed and photographed under electron microscope (JEM1400, Tokyo, Japan).

The criteria for distinguishing different parts are as follows: 1. Soma: Soma contains less heterochromatin, the color is light, and the nucleolus is large and obvious.The cytoplasm contains rough endoplasmic reticulum, free ribosome, Golgi complex, neurofilament and microtubule; 2. Mitochondrion: Mitochondrion is the organelle surrounded by two layers of membrane, which is oval or long strip, and its length changes greatly. 3. Dendrite: Dendrites contain Nissl body, mitochondria, Golgi complex, smooth endoplasmic reticulum, neurofilament and microtubule. Clusters of ribosomes often appear with irregular outline, prominent ratchet like accessory structures including hypertrophic mitochondria, microtubules, and post prominent specialized structures. 4. Axons: The axon structure is thin, smooth and without spines. Axons are usually separated from the cell body. Under the electron microscope, the main cell components in axons are free ribosome, mitochondria, neurofilament and microtubule. With the extension of axons, rough endoplasmic reticulum and ribosome gradually decreased or even disappeared. Some axons are surrounded by myelin sheath. 5. Axon terminals: The axon terminals are spherical, expanded and thickened to form presynaptic membrane. Synaptic vesicles and several mitochondria could be found in axon terminal.

### Glioma tissues screening and staining

#### Glioma tissues screening

Patient glioma tissues were provided by Xijing hospital. All patients provided written informed consent before glioma tissues recruitment. After screening, 3 cases of hairy cell astrocytoma, 6 cases of diffuse astrocytoma, 6 cases of anaplastic astrocytoma, 13 cases of glioblastoma, 13 cases of oligodendrocytoma, 13 cases of anaplastic oligodendrocytoma and 6 cases of paracancerous tissue were included in this study.

#### HE staining

HE staining was conducted according toroutine protocols. 4-μm sections were obtained from each paraffin block utilizing pathological microtome (Leica RM2235, Germany). After deparaffinization and rehydration, sections were stained with hematoxylin solution (Solarbio, G1080, China) for 5 mins, stained with eosin (Solarbio, G1100–100, China) for 3 mins and re-immersed in alcohol and xylene. The mounted slides were then examined and photographed using inverted microscope (NikonCI-S, Japan) and imaging system(NikonDS-U3, Japan).

#### Glioma staining

The slides of glioma cells were taken out from PBS and rinsed in PBS for 3 times at room temperature, 10 min/time; 10% calf serum was used for 2 h incubation; slides were incubated in anti-rabbit-anti-Drp1 (Abcam, ab184247, 1:1000, UK) overnight in a wet and dark box at room temperature; rinsed slices in PBS at room temperature for 3 times, 10 min/time in next day; incubated the slices in anti-a488-anti-rabbit (Abbkine, 1:500, USA) for 2 h; rinsed slices 3 times in PBS at room temperature, 10 min/time; sealed slices with fluorescent sealing agent and photographed under confocal microscope (FV1000, Olympus, Japan).

### Statistical analysis

Data are presented as the mean ± SEM (standard error of the mean). One-way repeated-measures ANOVA was used for the analysis of differences between the experimental groups. Kruskal-Wallis test was used to confirm the Drp1 distribution differences between dendrites, axons, axon terminals and somas under IEM. *p* < 0.05 was considered statistically significant. Data are analyzed through SPSS 21.0 software.

## Data Availability

The datasets used and analyzed during the current study are available from the corresponding author on reasonable request.

## Abbreviations

Drp1: Dynamin-related protein
GED: GTPase effector domain
GABA: γ-Aminobutyric acid
FISH: Fluorescence in situ hybridization
TEM: Transmission electron microscope
IEM: Immunoelectron microscopy
DMSO: Dimethylsulfoxide
PBS: Phosphate-buffered saline
SDS-PAGE: Sodium dodecyl sulfate-polyacry-lamide gel electrophoresis
SEM: Standard error of the mean
GAD67-GFP: Glutamic acid decarboxylase 67-green fluorescent protein
mito: Mitochondria
PAG: Periaqueductal gray
CTX: Cerebral cortex
cing: Cingulum bundle
SIMpu: Simple lobule, Purkinje cell
SIMmo: Simple lobule, molecular layer
SIMgr: Simple lobule, granular layer
cc: Corpus callosum
CA: Ammon’s horn
CA1-so: Field CA1, stratum oriens
CA1-sp: Field CA1, pyramidal layer
CA1-sr: Field CA1, stratum radiatum
CA1-slm: Field CA1, stratum lacunosum-moleculare
DG-mo: Molecule layer of dentate gyrus
DG-sg: Granular cell layer of dentate gyrus
DG-po: Polymorphic layer of dentate gyrus
LGd: Dorsal part of the lateral geniculate complex
LGv: Ventral part of the lateral geniculate complex
IGL: Intergeniculate leaflet of the lateral geniculate complex
MGv: Medial geniculate complex, ventral part
PB: Parabrachial nucleus
B: Barrington’s nucleus
LC: Locus ceruleus
MEV: Midbrain trigeminal nucleus
SUV: Superior vestibular nucleus
CN: Cochlear nuclei
VII: Facial motor nucleus
RSPd: Retrosplenial area, dorsal part
RSPv: Retrosplenial area, ventral part
PTLp: Posterior parietal association areas
AUD: Auditory areas
TEa: Temporal association areas
BLA: Basolateral amygdalar nucleus
PIR: Piriform area
alv: Alveus
df: Dorsal fornix
PO: Posterior complex of the thalamus
VPM: Ventral posteromedial nucleus of the thalamus
VPL: Ventral posterolateral nucleus of the thalamus
fr: Fasciculus retroflexus
PF: Parafascicular nucleus
IC: Inferior colliculus
AQ: Cerebral aqueduct
DR: Dorsal nucleus raphe
MRN: Midbrain reticular nucleus
V4: Fourth ventricle
PCG: Pontine central gray
scp: Superior cerebelar peduncles
V4r: Lateral recess
icp: Inferior cerebellar peduncle
FL: Flocculus
SPVO: Spinal nucleus of the trigeminal, oral part
sptV: Spinal tract of the trigeminal nerve
VCO: Ventral cochlear nucleus
DCO: Dorsal cochlear nucleus
IRN: Intermediate reticular nucleus
PARN: Parvicellular reticular nucleus
sctv: Ventral spinocerebellar tract
rust: Rubrospinal tract
SPVI: Spinal nucleus of the trigeminal, interpolar part
IP: Interposed nucleus
SPIV: Spinal vestibular nucleus
MDRNd: Medullary reticular nucleus, dorsal part
ECU: External cuneate nucleus
NTS: Nucleus of solitary tract
PAS: Parasolitary nucleus
NeuN: Neuronal nuclear antigen
NG: Nanogold particles
ODN: Oligodeoxynucleotide
LSD: Least significant difference

## References

[1] Reddy PH (2011). Dynamin-related protein 1 and mitochondrial fragmentation in neurodegenerative diseases. Brain Res Rev.

[2] Oliver D, Reddy PH. Dynamics of Dynamin-Related Protein 1 in Alzheimer’s Disease and Other Neurodegenerative Diseases. Cells. 2019;8(9). 10.3390/cells8090961 PMC6769467 31450774

[3] Ford MG, Jenni S, Nunnari J (2011). The crystal structure of dynamin. Nature.

[4] Smirnova E (2001). Dynamin-related protein Drp1 is required for mitochondrial division in mammalian cells. Mol Biol Cell.

[5] Wu W (2019). OPA1 overexpression ameliorates mitochondrial cristae remodeling, mitochondrial dysfunction, and neuronal apoptosis in prion diseases. Cell Death Dis.

[6] Gusdon AM (2017). Exercise increases mitochondrial complex I activity and DRP1 expression in the brains of aged mice. Exp Gerontol.

[7] Saxton WM, Hollenbeck PJ (2012). The axonal transport of mitochondria. J Cell Sci.

[8] Otera H, Ishihara N, Mihara K (2013). New insights into the function and regulation of mitochondrial fission. Biochim Biophys Acta.

[9] Wanders RJ, Waterham HR (2006). Biochemistry of mammalian peroxisomes revisited. Annu Rev Biochem.

[10] Ren X (2017). Resveratrol ameliorates mitochondrial elongation via Drp1/Parkin/PINK1 signaling in senescent-like cardiomyocytes. Oxidative Med Cell Longev.

[11] Cho B (2013). Physiological and pathological significance of dynamin-related protein 1 (drp1)-dependent mitochondrial fission in the nervous system. Exp Neurobiol.

[12] Qi Z (2019). Dynamin-related protein 1: a critical protein in the pathogenesis of neural system dysfunctions and neurodegenerative diseases. J Cell Physiol.

[13] Gao J, et al. Abnormalities of Mitochondrial Dynamics in Neurodegenerative Diseases. Antioxidants (Basel). 2017;**6**(2). 10.3390/antiox6020025 PMC5488005 28379197

[14] Gibellini L (2015). Natural compounds modulating mitochondrial functions. Evid Based Complement Alternat Med.

[15] Guo BL (2013). Significant changes in mitochondrial distribution in different pain models of mice. Mitochondrion.

[16] Ferrari LF (2011). Role of Drp1, a key mitochondrial fission protein, in neuropathic pain. J Neurosci.

[17] Dai CQ (2016). p53 and mitochondrial dysfunction: novel insight of neurodegenerative diseases. J Bioenerg Biomembr.

[18] Eugenio-Perez D (2019). Divide et Impera: Drp1-mediated mitochondrial fission in glioma malignancy. Yale J Biol Med.

[19] Michalska BM (2018). Insight into the fission mechanism by quantitative characterization of Drp1 protein distribution in the living cell. Sci Rep.

[20] D'Agata V (2002). Distribution of parkin in the adult rat brain. Prog Neuro-Psychopharmacol Biol Psychiatry.

[21] Reference Atlas*:: Allen Brain Atlas: Mouse brain.*. http://mouse.brain-map.org/ (2004). Accessed 25 Jan 2004.

[22] Fontes MAP (2018). GABA-containing liposomes: neuroscience applications and translational perspectives for targeting neurological diseases. Nanomedicine.

[23] Wong CG, Bottiglieri T, Snead OC (2003). GABA, gamma-hydroxybutyric acid, and neurological disease. Ann Neurol.

[24] Solas M, Puerta E, Ramirez MJ (2015). Treatment options in Alzheimer’s disease: the GABA story. Curr Pharm Des.

[25] Wang YY (2009). Expression patterns of 5-HT receptor subtypes 1A and 2A on GABAergic neurons within the spinal dorsal horn of GAD67-GFP knock-in mice. J Chem Neuroanat.

[26] Wang YY (2008). The effect of serotonin on GABA synthesis in cultured rat spinal dorsal horn neurons. J Chem Neuroanat.

[27] Bai Y (2018). Targeted upregulation of uncoupling protein 2 within the basal ganglia output structure ameliorates dyskinesia after severe liver failure. Free Radic Biol Med.

[28] Dykman LA (2018). Gold nanoparticles as an adjuvant: influence of size, shape, and technique of combination with CpG on antibody production. Int Immunopharmacol.

[29] El-Naggar ME (2016). Eco-friendly microwave-assisted green and rapid synthesis of well-stabilized gold and core-shell silver-gold nanoparticles. Carbohydr Polym.

[30] Zhou K (2019). Atractylenolide III ameliorates cerebral ischemic injury and neuroinflammation associated with inhibiting JAK2/STAT3/Drp1-dependent mitochondrial fission in microglia. Phytomedicine.

[31] Chae U (2019). Drp1-dependent mitochondrial fission regulates p62-mediated autophagy in LPS-induced activated microglial cells. Biosci Biotechnol Biochem.

[32] Itoh K, et al. Brain-specific Drp1 regulates postsynaptic endocytosis and dendrite formation independently of mitochondrial division. Elife. 2019;8. 10.7554/eLife.44739 PMC6824841 31603426

[33] Li Z (2004). The importance of dendritic mitochondria in the morphogenesis and plasticity of spines and synapses. Cell.

[34] Verstreken P (2005). Synaptic mitochondria are critical for mobilization of reserve pool vesicles at Drosophila neuromuscular junctions. Neuron.

[35] Rangaraju V, Lauterbach M, Schuman EM (2019). Spatially stable mitochondrial compartments fuel local translation during plasticity. Cell.

[36] Palmer CS (2011). MiD49 and MiD51, new components of the mitochondrial fission machinery. EMBO Rep.

[37] Lim TK (2015). Mitochondrial and bioenergetic dysfunction in trauma-induced painful peripheral neuropathy. Mol Pain.

[38] Joshi AU (2018). Drp1/Fis1 interaction mediates mitochondrial dysfunction, bioenergetic failure and cognitive decline in Alzheimer’s disease. Oncotarget.

[39] Fecher C (2019). Cell-type-specific profiling of brain mitochondria reveals functional and molecular diversity. Nat Neurosci.

[40] Molina V (2017). Cell cycle analysis in the rat external granular layer evaluated by several bromodeoxyuridine immunoperoxidase staining protocols. Histochem Cell Biol.

[41] Stumm RK (2004). Neuronal types expressing mu- and delta-opioid receptor mRNA in the rat hippocampal formation. J Comp Neurol.

[42] Ma JT (2019). Effects of dynamin-related protein 1 regulated mitochondrial dynamic changes on invasion and metastasis of lung Cancer cells. J Cancer.

[43] Aggarwal S (2019). Depletion of dAKAP1-protein kinase a signaling islands from the outer mitochondrial membrane alters breast cancer cell metabolism and motility. J Biol Chem.

[44] Tagaya M, Arasaki K (2017). Regulation of mitochondrial dynamics and autophagy by the mitochondria-associated membrane. Adv Exp Med Biol.

[45] Santel A, Frank S (2008). Shaping mitochondria: the complex posttranslational regulation of the mitochondrial fission protein DRP1. IUBMB Life.

[46] Chang CR, Blackstone C (2007). Drp1 phosphorylation and mitochondrial regulation. EMBO Rep.

[47] Song Y (2019). Inhibition of Drp1 after traumatic brain injury provides brain protection and improves behavioral performance in rats. Chem Biol Interact.

[48] Kanda H (2016). Inhibition of mitochondrial fission protein reduced mechanical allodynia and suppressed spinal mitochondrial superoxide induced by Perineural human immunodeficiency virus gp120 in rats. Anesth Analg.

[49] Zhou K (2017). RIP1-RIP3-DRP1 pathway regulates NLRP3 inflammasome activation following subarachnoid hemorrhage. Exp Neurol.

[50] Wu Q (2016). Mitochondrial division inhibitor 1 (Mdivi-1) offers neuroprotection through diminishing cell death and improving functional outcome in a mouse model of traumatic brain injury. Brain Res.

[51] Tamamaki N (2003). Green fluorescent protein expression and colocalization with calretinin, parvalbumin, and somatostatin in the GAD67-GFP knock-in mouse. J Comp Neurol.

[52] Zimmermann M (1983). Ethical guidelines for investigations of experimental pain in conscious animals. Pain.

[53] Lein ES (2007). Genome-wide atlas of gene expression in the adult mouse brain. Nature.

[54] Paxinos, G. and K.B.J. Frankin, *The Mouse Brain in Stereotaxic Coordinates*. 2nd ed. ACADEMIC PRESS; 1997.

[55] Crossman, A.R. and D. Neary, *Neuroanatomy: An Illustrated Colour Text*. 5th ed. Elsevier Limited; 2015.

[56] Luo TT (2017). Distribution of mitochondrial dynamin-related protein 1 mRNAs in amygdala complex of mice based on the FISH techenique. Chin J Neuroanat.

[57] Yang YL (2013). Abnormal chloride homeostasis in the substancia nigra pars reticulata contributes to locomotor deficiency in a model of acute liver injury. PLoS One.

